# Effects of chronic insecticide exposure on neuronal network development in vitro in rat cortical cultures

**DOI:** 10.1007/s00204-024-03840-0

**Published:** 2024-08-20

**Authors:** Lennart V. J. van Melis, Anneloes M. Peerdeman, Celia Arenas González, Regina G. D. M. van Kleef, J. Pepijn Wopken, Remco H. S. Westerink

**Affiliations:** Neurotoxicology Research Group, Division of Toxicology, Faculty of Veterinary Medicine, Institute for Risk Assessment Sciences (IRAS), Utrecht University, P.O. Box 80.177, NL-3508 TD Utrecht, The Netherlands

**Keywords:** Pesticides, Micro-electrode array (MEA), Neuronal network activity, Sex-specific effects, Developmental neurotoxicity (DNT)

## Abstract

Developmental exposure to carbamates, organophosphates, and pyrethroids has been associated with impaired neurodevelopmental outcomes. Sex-specific differences following chronic insecticide exposure are rather common in vivo. Therefore, we assessed the chronic effects of in vitro exposure to different carbamates (carbaryl, methomyl and aldicarb), organophosphates [chlorpyrifos (CPF), chlorpyrifos-oxon (CPO), and 3,5,6,trichloropyridinol (TCP)], and pyrethroids [permethrin, alpha-cypermethrin and 3-phenoxy benzoic acid (3-PBA)] on neuronal network development in sex-separated rat primary cortical cultures using micro-electrode array (MEA) recordings. Our results indicate that exposure for 1 week to carbaryl inhibited neurodevelopment in male cultures, while a hyperexcitation was observed in female cultures. Methomyl and aldicarb evoked a hyperexcitation after 2 weeks of exposure, which was more pronounced in female cultures. In contrast to acute MEA results, exposure to ≥ 10 µM CPF caused hyperexcitation in both sexes after 10 days. Interestingly, exposure to 10 µM CPO induced a clear hyperexcitation after 10 days of exposure in male but not female cultures. Exposure to 100 µM CPO strongly inhibited neuronal development. Exposure to the type I pyrethroid permethrin resulted in a hyperexcitation at 10 µM and a decrease in neuronal development at 100 µM. In comparison, exposure to ≥ 10 µM of the type II pyrethroid alpha-cypermethrin decreased neuronal development. In female but not in male cultures, exposure to 1 and 10 µM permethrin changed (network) burst patterns, with female cultures having shorter (network) bursts with fewer spikes per (network) burst. Together, these results show that MEA recordings are suitable for measuring sex-specific developmental neurotoxicity in vitro. Additionally, pyrethroid exposure induced effects on neuronal network development at human-relevant concentrations. Finally, chronic exposure has different effects on neuronal functioning compared to acute exposure, highlighting the value of both exposure paradigms.

**Supplementary Information:**

The online version contains supplementary material available at 10.1007/s00204-024-03840-0.

## Introduction

During a lifetime people are exposed to numerous insecticides via food, water and air. Exposure to these insecticides has been linked to various health problems. Previously, we showed the acute neurotoxic effects of carbamates, organophosphates, and pyrethroids, which for some insecticides resulted in sex-specific effects (van Melis et al. 2024). However, chronic exposure is more human-relevant (Costa et al. 2008) and has been associated with impaired neurodevelopmental outcomes (Flaskos 2012; Grandjean and Landrigan 2014; Lee et al. 2015). Moreover, sex-specific differences following chronic insecticide exposure are rather common (Kern et al. 2017; Wagner-Schuman et al. 2015; Wang et al. 2023). It is therefore of interest to investigate the possible sex-specific effects of chronic insecticide exposure on neuronal network development.

The proliferation of neural stem cells, migration, neuronal and glial cell differentiation, formation of neurites and synapses, myelination, and neuronal network formation and function are all key events in human brain development (Rice and Barone 2000; Stiles and Jernigan 2010) and are dependent on the interplay between signaling factors and excitatory/inhibitory balance. Animal studies have shown that exposure to environmental chemicals during critical periods can disrupt brain development, leading to adverse health effects (Grandjean and Landrigan 2006). Disruption of these neurodevelopmental processes are also described as common key events in adverse outcome pathway (AOP) networks for human developmental neurotoxicity (Spinu et al. 2019). Studies have shown that males and females can differ in brain volume, fiber density, synapses, branching, and cell numbers (see McCarthy et al. 2017 for an overview). Furthermore, sex-specific differences are found in the expression of glutamatergic AMPA and NMDA receptors (Brandt et al. 2020; Knouse et al. 2022, 2023), voltage-gated calcium channels (Chang et al. 2007) and the number of GABAergic neurons (Galanopoulou 2008). During development, GABA receptor signaling switches from depolarizing to hyperpolarizing, known as the GABA switch. This switch occurs earlier in females than males (Wolf et al. 2022). Finally, expression of brain-derived neurotrophic factor (BNDF), which promotes neuronal survival, differentiation, neurite outgrowth, and synapse formation (Numakawa et al. 2010; Stansfield et al. 2012), is higher in females than males (Wolf et al. 2022). The sex-specific differences during neurodevelopment are not only regulated by steroid hormones but also by genetic differences (Paylar et al. 2024; Vigé et al. 2008). As a result, there are sensitive periods during development in which chemical insults have different effects between males and females.

Chronic exposure to organophosphates can inhibit axonal transport (Naughton and Terry 2018; Richardson et al. 2019), decrease neurite outgrowth (Das and Barone 1999), and change the expression of genes involved in neurotransmission (Savy et al. 2018). Exposure to carbamates can change levels of proteins that are important in neuronal development, such as CaM kinase II, GAP-43, and tau (Lee et al. 2015). Studies in rats have shown that motor coordination was impaired by developmental exposure to the organophosphate chlorpyrifos and the pyrethroid cypermethrin in females, but not males. Chlorpyrifos increased motor activity in both sexes, while cypermethrin decreased motor activity mainly in males (Gómez-Giménez et al. 2018). Also, chronic exposure to chlorpyrifos and cypermethrin impaired learning in males, but not in females (Gómez-Giménez et al. 2017). Exposure to cypermethrin reduced neuronal proliferation, maturation, and differentiation in mice (Guo et al. 2018). Exposure to another pyrethroid, deltamethrin, changed NMDA receptor levels in the hippocampus of male, but not female rats. Moreover, long-term potentiation was increased in male rats (Pitzer et al. 2022). Developmental exposure to deltamethrin resulted in long-term down-regulation of voltage-gated sodium channel mRNA expression in mice, which could lead to persistent changes in neuronal function (Magby and Richardson 2017). Studies using multi-well microelectrode arrays (MEA) showed that chlorpyrifos inhibits neuronal activity during both acute and chronic exposure, while chlorpyrifos-oxon has effects following acute, but not during chronic exposure. This suggests an adaptive capacity in the developing neuronal network (Dingemans et al. 2016; Richardson et al. 2019). Chronic exposure for 14 days to cypermethrin also inhibited the development of spontaneous neuronal activity, while exposure to carbaryl had no effect (Dingemans et al. 2016).

These studies show that chronic exposure to insecticides can have sex-specific effects on neuronal development in vivo and in vitro, and previous studies using MEA recordings have shown that some of these insecticides also affect neuronal activity and neuronal network functioning. However, to our knowledge, the sex-specific effects of chronic insecticide exposure on neuronal network functioning have not yet been studied in vitro. The present study therefore aimed to determine the sex-specific effects of chronic exposure to insecticides using MEA recordings, which enable the recording of spontaneous neuronal activity. Neuronal networks grown on an MEA develop spontaneous activity over time and are responsive to a variety of drugs and chemicals, including insecticides (Kosnik et al. 2020; Strickland et al. 2018; van Melis et al. 2023, 2024). Since MEA recordings are non-invasive, this allows for multiple recordings over time, so that the development of neuronal network function can be followed (Johnstone et al. 2010; Mack et al. 2014). Acute neurotoxicity, measured using MEA recordings, may arise from an acute disturbance of calcium homeostasis, receptor and ion channel function, and/or synaptic communication (Gerber et al. 2021; Johnstone et al. 2010; Vassallo et al. 2017). The effects of acute insecticide exposure on neuronal activity were shown in a previous study (van Melis et al. 2024). Chronic exposure studies allow us to not only examine these immediate effects, but also enable analysis of changes in network integrity and cell viability. Additionally, this design facilitates investigation into the reversibility of acute neurotoxic effects through adaptive changes. The effects found after chronic exposure could be related to changes in the expression of ion channels and neurotransmitter receptors, AMPA- or NMDA receptor trafficking, changes in synaptic density, and growth or retraction of axons and dendrites, resulting in changes in plasticity. Finally, our chronic exposure design starts when spontaneous neuronal (network) activity has started, but is still maturing. This enables us to investigate the effects of chronic exposure on a developing neuronal network rather than a fully mature one, as typically observed in acute exposure studies. Primary rat cortical cultures are the current standard for MEA recordings (Hondebrink et al. 2016; Tukker et al. 2020). By sex-separating these cultures, it is possible to measure sex-specific effects on neuronal network development in vitro (van Melis et al. 2024). Here, we investigated the effects of chronic exposure to several carbamates (aldicarb, carbaryl, and methomyl), organophosphates (chlorpyrifos, chlorpyrifos-oxon, and 3,5,6-trichloropyridinol (TCP)), and pyrethroids (permethrin, alpha-cypermethrin and 3-phenoxy-benzoic acid (3-PBA)) on neuronal network development in in vitro rat primary cortical cultures.

## Methods

### Chemicals

Chlorpyrifos oxon (purity 98.7%) was obtained from AccuStandard (New Haven, CT, USA). Aldicarb (purity 99.9%) and carbaryl (purity 99.7%) were obtained from Riedel-de Haën (Seelze, Germany). 3-phenoxy-benzoic acid (3-PBA; purity 99.7%) was obtained from Dr. Ehrenstorfer (Augsburg, Germany). Phenol-red free neurobasal-A (NB-A) medium, l-glutamine (200 mM), glutamate (3.5 mM) penicillin/streptomycin (5000 U/mL/5000 mg/mL), and B-27 plus supplement were purchased from Life Technologies (Bleiswijk, the Netherlands). Unless otherwise noted, all other chemicals were obtained from Sigma-Aldrich (Zwijndrecht, the Netherlands). Pesticide stock solutions of 10^–1^–10^–5^ M were prepared in dimethyl sulfoxide (DMSO) and diluted in culture medium just before the experiments. All solutions used in experiments, including control experiments, contained 0.1% DMSO.

### Cell culture

Sex-specific, primary cultures of rat cortical neurons were prepared from pups born of timed-pregnant Wistar rats (Envigo, Horst, the Netherlands) on postnatal day 0 or 1 as described previously (Gerber et al. 2021). Briefly, rat pups were sexed by looking at the anogenital distance and separated between males and females. Next, pups were decapitated and the cortices were isolated and placed in ice-cold dissection medium (450 mL NBA medium, 14 g sucrose, 1.25 mL l-glutamine (200 mM), 5 mL glutamate (3.5 mM), 5 mL penicillin/streptomycin and 10 mL B-27, pH 7.4). Cortices were minced and triturated to a homogenous suspension and filtered through an easy strainer (100 μm, Greiner Bio One, Alphen aan den Rijn, The Netherlands). Subsequently, cells were centrifuged for 5 min at 800 rpm. The supernatant was removed and the pellet was resuspended using 1 mL of dissection medium per rat brain and diluted to a cell suspension containing 2 × 10^6^ cells/mL. Next, drops (50 µL/well) of cell suspension were seeded at a density of 1 × 10^5^ cells/well on 48-well microelectrode array (MEA) plates (Axion Biosystems Inc., Atlanta, GA, USA) coated with PEI [0.1% PEI solution in borate buffer (24 mM sodium borate/50 mM boric acid in Milli-Q adjusted to pH 8.4)]. Cells were allowed to attach in a humidified 5% CO_2_/95% air atmosphere for 2 h at 37 °C before 450 µL dissection medium was added to each well. At 4 days in vitro (DIV 4), 450 µL dissection medium was replaced by 450 µL glutamate-free medium (450 mL NBA medium, 14 g sucrose, 1.25 mL l-glutamine (200 mM), 5 mL penicillin/streptomycin, and 10 mL B-27, pH 7.4). Cells were cultured in 5% CO_2_/95% air atmosphere at 37 °C until use at DIV 9–11.

All animal experiments were performed in agreement with Dutch law, and the European Community directives regulating animal research (2010/63/EU) and approved by the Ethical Committee for Animal Experiments of Utrecht University. All efforts were made to minimize the number of animals used and their suffering.

### MEA recordings

Multi-well MEA plates were used to record spontaneous neuronal activity. These plates contain 48 wells per plate, each well containing an array of 16 individual embedded nanotextured microelectrodes (40–50 µm diameter; 350 µm center-to-center spacing), yielding a total of 768 channels, which can be recorded at the same time. Recordings were made as previously described (Gerber et al. 2021). Given human exposure estimates, concentrations > 10 µM generally lack toxicological relevance even when taking inter- and intraspecies uncertainty factors into account. Hence all compounds were tested at final concentrations of 0.1–100 µM. Each well was exposed to only one condition (i.e., one concentration of one test compound) to prevent the potential effects of cumulative dosing.

For each recording, a 48-well MEA plate was placed in a Maestro 768-channel amplifier with an integrated heating system (set at 37 °C), temperature controller, and data acquisition interface (Axion Biosystems Inc., Atlanta, GA, USA). Before each recording, MEA plates were allowed to equilibrate for around 5 min, after which a 30-min recording of spontaneous activity was started. Neuronal activity was recorded from DIV 0 to DIV 28 with exposure to DMSO (solvent control) to characterize normal neuronal development. Based on these results and previous studies (Dingemans et al. 2016), DIV 7 was chosen as the first measurement day for experiments with exposure to carbamates, organophosphates, and pyrethroids. At DIV 7, neuronal networks have started developing, and sufficient spontaneous network activity can be measured. Wells with at least four bursting electrodes at baseline recording (recording at DIV 7) were included in experiments. After the baseline recording, a half-medium change was performed to expose the cells to the desired concentration of the test compound. During the half-medium change, the medium was first resuspended, after which half the medium (250 µL) was removed. This was replaced by 250 µL fresh medium containing the desired exposure concentration of the test compound. At DIV 10, 14, 17, 21, and 24 another 30 min MEA recording was performed, followed by a half-medium change. After the final MEA measurement on DIV 28, cell viability was assessed using the Alamar Blue assay.

### Cell viability assay

During the chronic MEA assay, the cells were exposed to a range of concentrations up to 100 µM for 21 days (DIV 7–28). After the final MEA measurement on DIV 28, the same cells were used to assess the effects of the different compounds on cell viability. Cell viability was assessed using the Alamar Blue (AB) assay (protocol adapted from Bopp and Lettieri 2008). Mitochondrial activity of the cells was recorded as a measure of cell viability with the AB assay, which is based on the ability of the cells to reduce resazurin to resorufin. Briefly, following exposure, cells were incubated for 75 min with 12.5 µM AB solution in HBSS (Invitrogen, Breda, The Netherlands) in the 48-wells MEA plate. Resorufin was measured spectrophotometrically at 540/590 nm (Infinite M200 microplate; Tecan Trading AG, Männedorf, Switzerland).

### Data analysis and statistics

Data analysis for the MEA data was performed as described in (Gerber et al. 2021). Briefly, MEA data acquisition was managed with Axion’s Integrated Studio (AxIS version 2.6). Raw data files were obtained by sampling channels simultaneously with a gain of 1200 × and a sampling frequency of 12.5 kHz/channel using a band-pass filter (200–5000 Hz).

Raw data were pre-processed to obtain.spk files. Spikes were detected using the AxIS spike detector (Adaptive threshold crossing, Ada BandFlt v2) with a post/pre-spike duration of 3.6/2.4 ms and a spike threshold of 7 × SD of the internal noise level (rms) of each individual electrode. Spike information was then further analyzed using NeuralMetrics Tool (v3.1.7, Axion BioSystems) and custom-made macros in Excel. Electrodes that recorded ≥ 6 spikes per minute were included in the analysis. A burst is a cluster of spikes measured on a single electrode. Bursts were defined using the Poisson surprise method (Legendy and Salcman 1985) with a minimum of 10 surprises. A network burst is a coordinated cluster of spiking across multiple electrodes. Network bursts were defined using an adaptive threshold with a minimum of 40 spikes, each separated by a maximum interval set automatically on a well-by-well basis based on the mean spike rate of each well, for a minimum of 15% of the electrodes/well. Data from the last 20 min of the 30-min recording were used for analysis since this is the most stable timeframe (see Hondebrink et al. 2016).

Based on an earlier principal component analysis (PCA; see van Melis et al. 2024), the ten most important MEA parameters were determined (see Supplementary data, table S1). These are number of spikes, number of (network) bursts, inter-burst interval, (network) burst duration, mean inter-spike interval (ISI) within network bursts, number of spikes per (network) burst, and area under cross-correlation, a measure for synchronicity.

To characterize normal neuronal development, and to test if cultures derived from males or females differed in their basal neuronal development over time, primary cultures originating from at least two different isolations were used. The data represent average values from DIV 0 to DIV 28 derived from 46 to 67 wells (n) from 2 to 3 (N) independent experiments and are presented as mean % ± standard error of the mean (SEM). Statistical analyses were performed using GraphPad Prism v9.2.0 (GraphPad Software, San Diego, CA, USA) using one-way ANOVA with Tukey post-hoc tests. A p-value ≤ 0.05 was considered statistically significant to determine sex-specific effects.

For the test compounds, first, raw values per parameter from all wells were grouped by condition. Treatment ratios for each parameter were calculated by using a cumulative approach, in which the raw values per well were added to each other to get a cumulative value. Next, the cumulative values from all conditions were normalized to the mean cumulative control value at DIV 28. Experimental values that exceeded mean ± 2 × SD (of their respective condition) were considered to be outliers (3.9% outliers) and therefore excluded from further analysis. This results in a control developmental curve that ends at a treatment ratio of 100% on DIV 28, reflecting baseline development. For the different conditions, the developmental curve can be above or below the control curve, reflecting a hyper- and hypo-excitation, respectively.

For each experimental condition, primary cultures originating from at least three different isolations were used. The data represent average values derived from 12 to 32 wells (*n*) from 2 to 5 (N) independent experiments and are presented as mean % ± standard error of the mean (SEM). Statistical analyses were performed using GraphPad Prism v9.2.0 (GraphPad Software, San Diego, CA, USA) using one-way ANOVA (cell viability) and two-way ANOVA (two factors; concentration and sex) with Tukey post-hoc tests. A p-value ≤ 0.05 was considered statistically significant. Sex-specific effects are shown as open symbols in the graphs and highlighted in the panels on the right side of each graph. Benchmark response (BMR) cut-offs were based on the average variation in all pooled DMSO control experiments. Effects that are smaller than the BMR are considered to be of limited toxicological relevance and are not indicated with an asterisk in the graphs, even if they are statistically significantly different from the time-matched control.

## Results

**Fig. 1:**
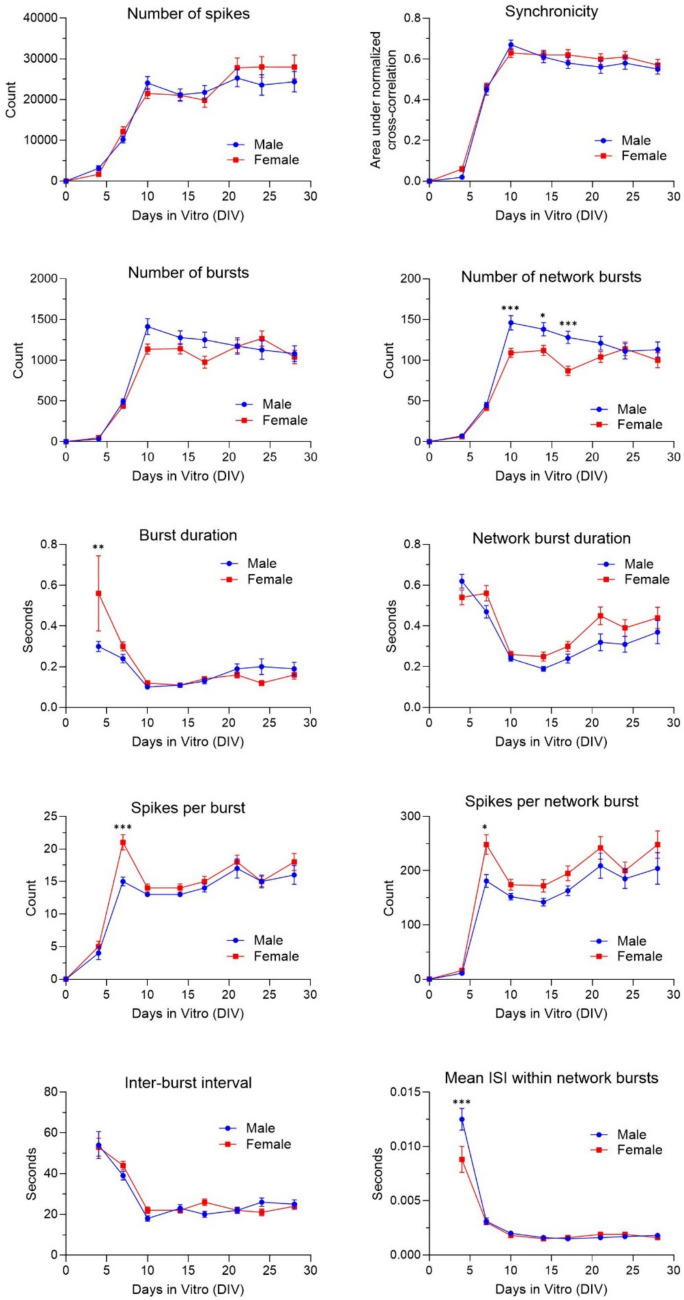
Average neuronal activity ± SEM from 46 to 67 individual wells (2–3 independent cultures) in male and female cultures during 28 days of development with exposure to DMSO (solvent control) from DIV 0 to 28. Differences between sexes (*p ≤ 0.05; **p ≤ 0.01; ***p ≤ 0.001)

To characterize normal neuronal development, and to test if cultures derived from males or females differed in their basal neuronal development over time, we compared the mean number of spikes, number of (network) bursts, (network) burst duration, number of spikes per (network) burst, mean ISI within network bursts and synchronicity between male (*n* = 46 wells; N = 2 independent experiments) and female (*n* = 67 wells; N = 2 independent experiments) cultures during 28 days (with exposure to DMSO (solvent control) from DIV 0–28). Neuronal cultures started to become active at DIV 4, with all wells showing spontaneous activity on DIV 7. The neuronal network reached a high, stable level of activity around DIV 10. Although there are some transient effects on DIV 4 and 7, the only profound differences between male and female cultures were found in the number of network bursts (higher in males from DIV 10 – 17; F (8, 1016) = 3.16), with all other parameters showing no clear differences (Fig. 1).

Although some activity was observed at DIV 4, the amount of activity at this time point was very low, making the quantification of the effects observed at DIV 4 unreliable. Based on these activity levels, and previous studies (Dingemans et al. 2016), DIV 7 was chosen as the time point to start exposure to carbamates, organophosphates, and pyrethroids and to perform the first measurement to investigate the effects of chronic insecticide exposure.

### Carbamates

**Fig. 2:**
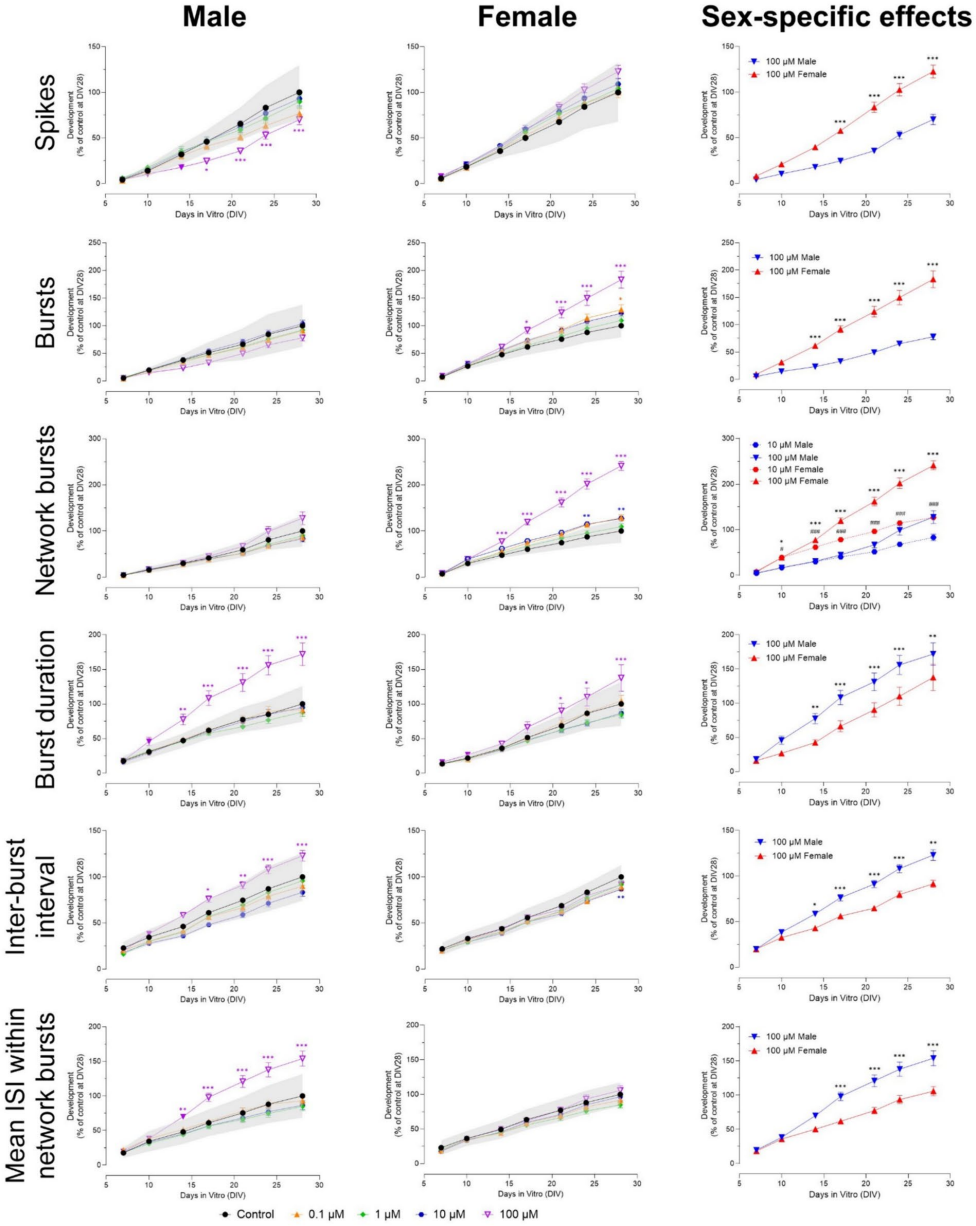
Exposure to carbaryl (DIV 7–28) affects a subset of neuronal activity parameters in both male (left panel) and female (middle panel) cultures. The grey shaded area represents a benchmark response derived from the variation in DMSO control experiments. Data points display the average percentage compared to control (DMSO control at DIV 28 set to 100%) ± SEM from 12–16 individual wells (≥ 2 independent experiments per concentration). Difference from DMSO control (*p ≤ 0.05; **p ≤ 0.01; ***p ≤ 0.001). The color of asterisks indicates which concentration is significantly affected. Differences between sexes are depicted as open symbols. Exposure to carbaryl evokes sex-specific effects at 10 and 100 µM (right panel). Sex-specific differences (*/#*p* ≤ 0.05; **/##*p* ≤ 0.01; ***/###*p* ≤ 0.001) are depicted with hashtags for 10 µM and asterisks for 100 µM

Exposure to carbaryl affected neuronal development from ≥ 10 µM. The number of spikes decreased in male but not in female cultures (Figs. 2 and S1) from DIV 17 to 28. This difference between male and female cultures was significant (F (54, 1016) = 1.97). Female but not male cultures showed an increase in the number of bursts (from DIV 17 to 28) and network bursts (from DIV 14–28; Figs. 2 and S1). Sex-specific effects were found at 100 µM for bursts (F (54, 998) = 4.86) and ≥ 10 µM for network bursts (F (54, 1023) = 13.2). Network burst duration was not affected in either sex (Fig. S2), but burst duration increased in both sexes, with a more profound and earlier increase in male cultures (Fig. 2; F (54, 990) = 2.86). The number of spikes per network burst decreased from DIV 14–28 in both sexes, but only reached significance in male cultures (Fig. S2). This effect was not sex-specific. The number of spikes per burst was not affected in either sex (Fig. S2). Inter-burst interval and mean ISI within network bursts increased from DIV 14–28 in male but not in female cultures (Fig. 2; F (54, 1011) = 1.95 for inter-burst interval, F (54, 1015) = 3.17 for mean ISI within network bursts). Synchronicity was not affected by exposure to carbaryl (Fig. S2).

**Fig. 3:**
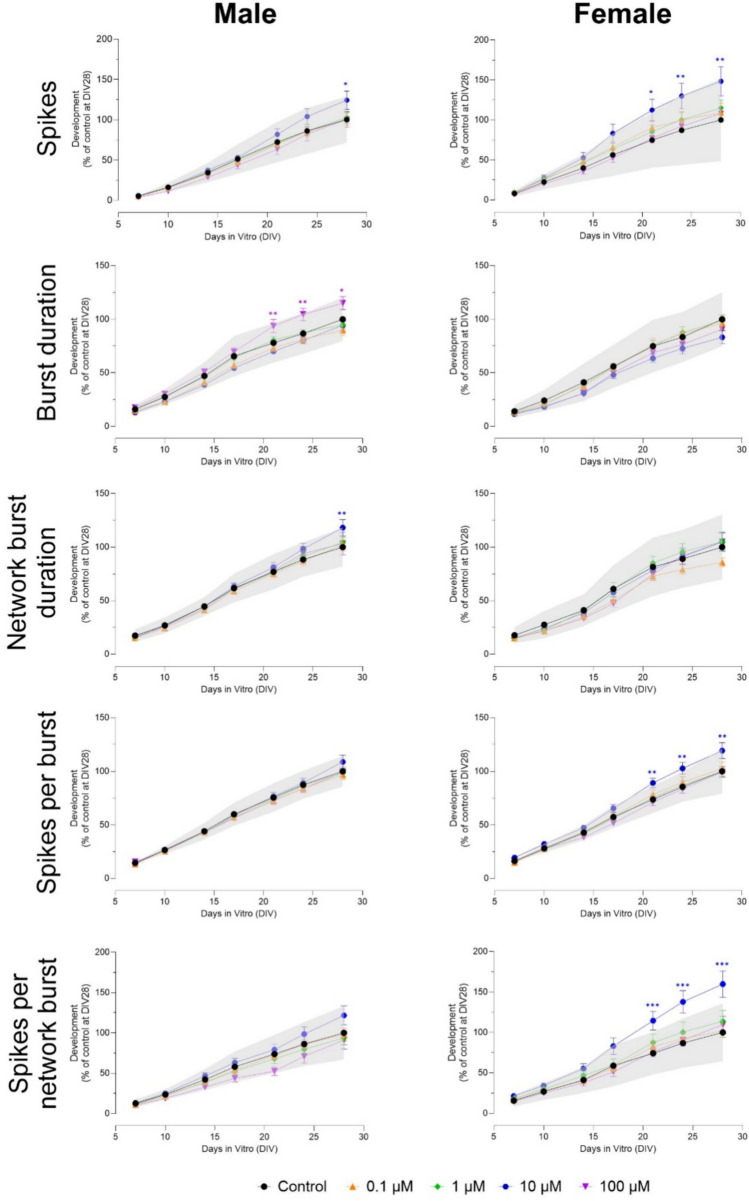
Exposure to aldicarb (DIV 7–28) affects a subset of neuronal activity parameters in both male (left) and female (right) cultures. The grey shaded area represents a benchmark response derived from the variation in DMSO control experiments. Data points display the average percentage compared to control (DMSO control at DIV 28 set to 100%) ± SEM from 14–22 individual wells (≥ 3 independent experiments per concentration). Difference from DMSO control (*p ≤ 0.05; **p ≤ 0.01; ***p ≤ 0.001). The color of asterisks indicates which concentration is significantly affected

Exposure to 10 µM aldicarb increased the number of spikes in male (only at DIV 28) and female (from DIV 21 to 28) cultures (Fig. 3), while no effect was found on the number of (network) bursts (Fig. S3). Network burst duration was increased in male (only at DIV 28) but not in female cultures. Exposure to 100 µM aldicarb increased burst duration in male cultures from DIV 21–28, but had no effect in female cultures (Fig. 3). The number of spikes per (network) burst was increased after exposure to 10 µM in female, but not in male cultures from DIV 21–28 (Fig. 3). Inter-burst interval was not affected by either sex (Fig. S3). Mean ISI within network bursts was increased from DIV 21 to 28 in male cultures after exposure to 100 µM, but decreased from DIV 17 to 28 in female cultures after exposure to 10 µM (Fig. S3). Synchronicity increased after exposure to 10 µM aldicarb in both male and female cultures, but only at DIV 28 (Fig. S3). None of these effects were sex-specific.

**Fig. 4:**
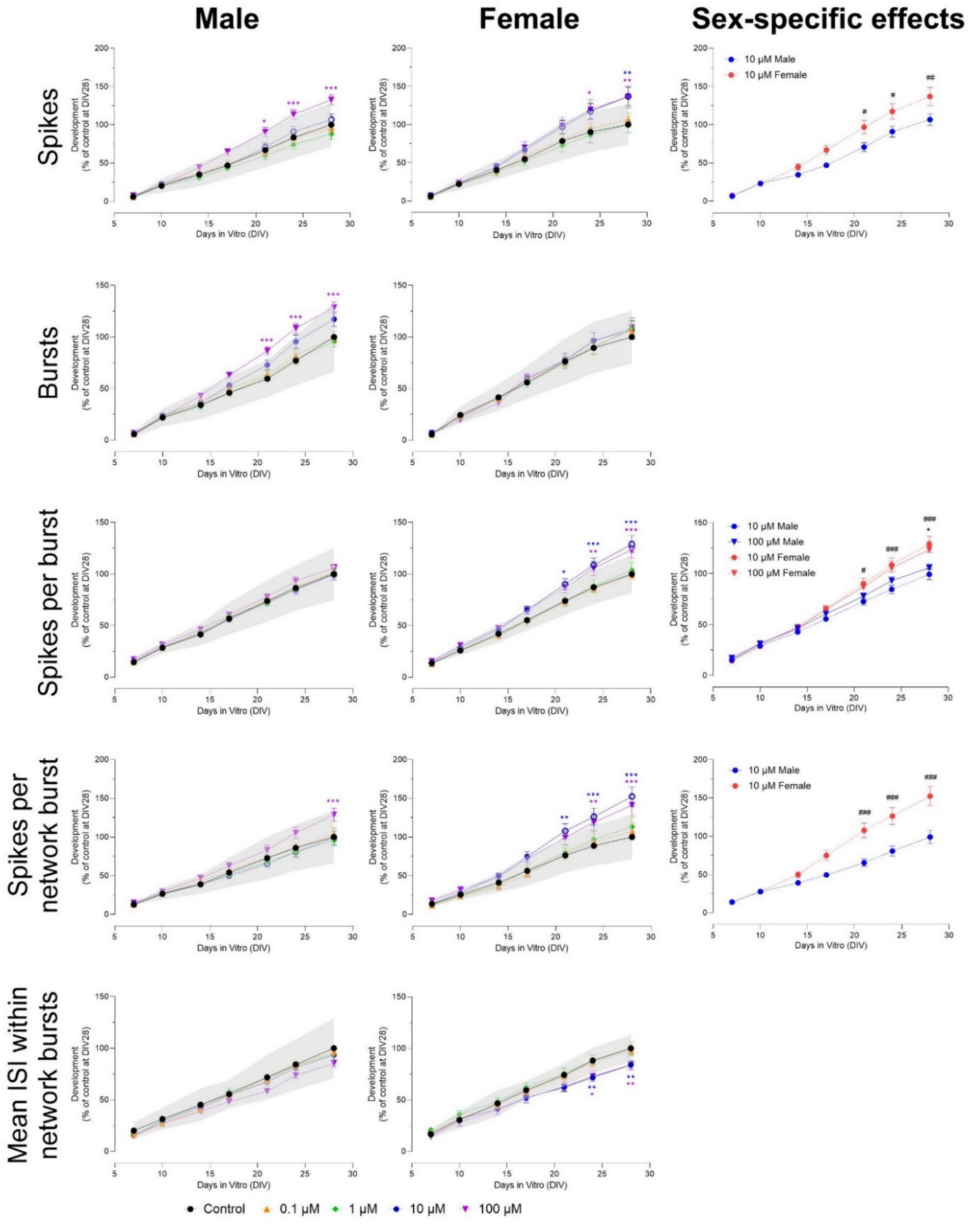
Exposure to methomyl (DIV 7–28) affects a subset of neuronal activity parameters in both male (left panel) and female (middle panel) cultures. The grey shaded area represents a benchmark response derived from the variation in DMSO control experiments. Data points display the average percentage compared to control (DMSO control at DIV 28 set to 100%) ± SEM from 16 to 21 individual wells (≥ 3 independent experiments per concentration). Difference from DMSO control (* p ≤ 0.05; ** *p* ≤ 0.01; *** *p* ≤ 0.001). The color of asterisks indicates which concentration is significantly affected. Differences between sexes are depicted as open symbols. Exposure to methomyl evokes sex-specific effects at 10 and 100 µM (right panel). Sex-specific differences (*/# *p* ≤ 0.05; **/## *p* ≤ 0.01; ***/### *p* ≤ 0.001) are depicted with hashtags for 10 µM and asterisks for 100 µM

Exposure to 100 µM methomyl increased the number of spikes in both male (from DIV 21–28) and female (from DIV 24 to 28) cultures (Fig. 4). Exposure to 10 µM induced a sex-specific effect and increased the number of spikes in female, but not in male cultures (F (54, 1224) = 1.58). The number of bursts was increased from DIV 21 to 28 in male, but not in female cultures (Fig. 4). This effect was not sex-specific. The number of network bursts was not affected in either sex (Fig. S4). The number of spikes per burst increased in female, but not male cultures from DIV 21 to 28 after exposure to 10 and 100 µM (Fig. 4). This effect was sex-specific (F (54, 1239) = 1.41). The number of spikes per network burst increased in male cultures after exposure to 100 µM (only at DIV 28) and in female cultures from DIV 21 to 28 (Fig. 4). Exposure to 10 µM evoked a sex-specific effect and increased the number of spikes per network bursts in female, but not in male cultures (F (54, 1236) = 1.61). (Network) burst duration was not affected in both sexes (Fig. S4). Inter-burst interval was decreased in male cultures from DIV 21–28 after exposure to 100 µM (Fig. S4). Mean ISI within network bursts was decreased in female cultures from DIV 24 to 28 after exposure to ≥ 10 µM methomyl (Fig. 4). Synchronicity was not affected by exposure to methomyl (Fig. S4).

Concluding, exposure to ≥ 10 µM carbaryl had sex-specific effects that started to appear after 1 week of exposure. In male cultures, neuronal development was delayed, while in female cultures, a hyperexcitation was observed. Exposure to ≥ 10 µM aldicarb resulted in hyperexcitation that seems to be more pronounced in female than in male cultures, but sex-specific effects were not found. Exposure to 100 µM methomyl also caused a hyperexcitation. Exposure to 10 µM methomyl had sex-specific effects, with a hyperexcitation in female but not in male cultures. The effects found after aldicarb and methomyl exposure only appeared after 2 weeks of exposure. Exposure to carbaryl, aldicarb, and methomyl did not affect cell viability (Fig. S5).

### Organophosphates

**Fig. 5:**
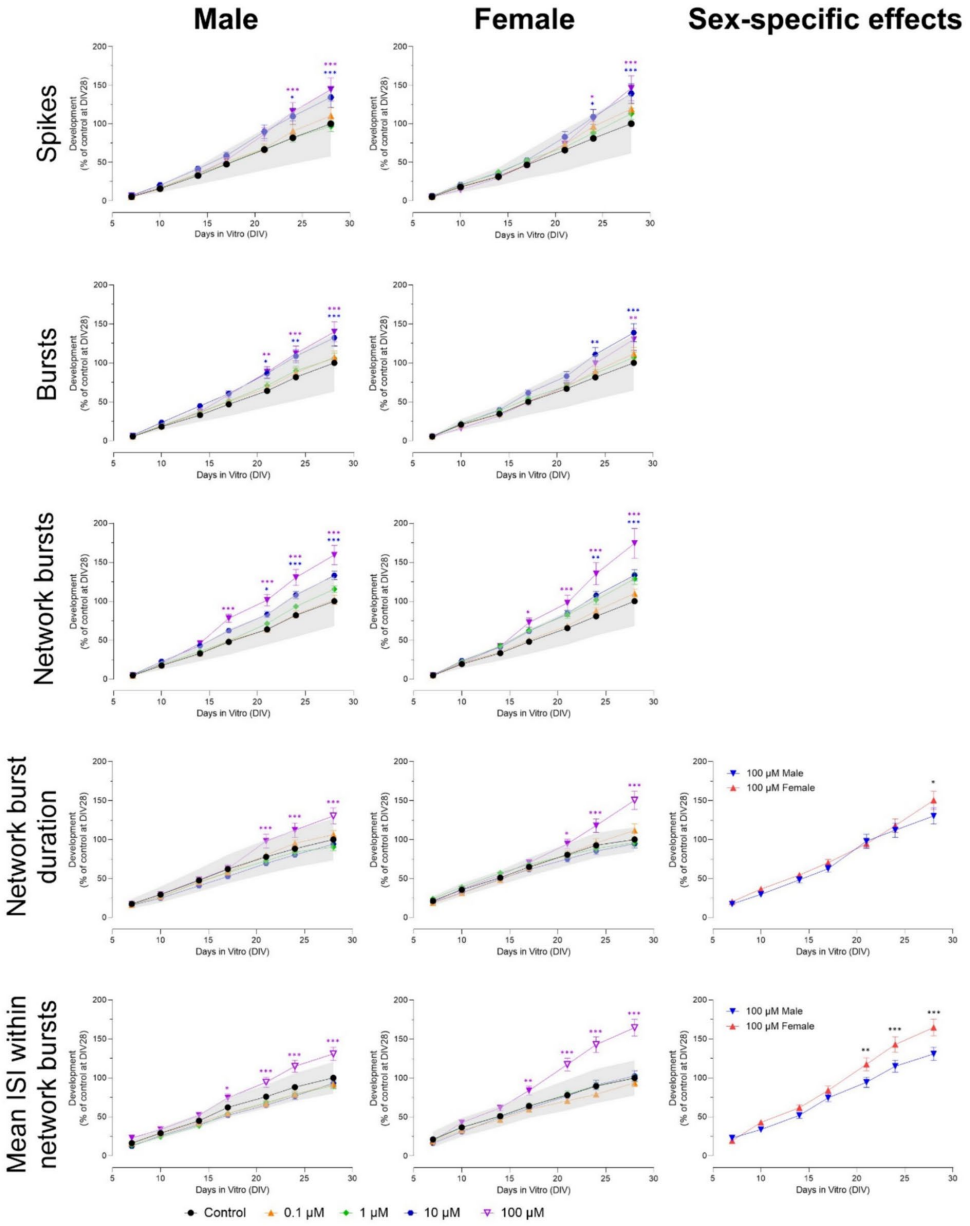
Exposure to chlorpyrifos (DIV 7–28) affects a subset of neuronal activity parameters in both male (left panel) and female (middle panel) cultures. The grey shaded area represents a benchmark response derived from the variation in DMSO control experiments. Data points display the average percentage compared to control (DMSO control at DIV 28 set to 100%) ± SEM from 18 to 32 individual wells (≥ 5 independent experiments per concentration). Difference from DMSO control (*p ≤ 0.05; **p ≤ 0.01; ***p ≤ 0.001). The color of asterisks indicates which concentration is significantly affected. Differences between sexes are depicted as open symbols. Exposure to chlorpyrifos evokes sex-specific effects at 100 µM (right panel). Sex-specific differences are depicted with asterisks (*p ≤ 0.05; **p ≤ 0.01; ***p ≤ 0.001)

Exposure to ≥ 10 µM chlorpyrifos increased the number of spikes (from DIV 24 to 28), number of bursts (from DIV 21 to 28), and number of network bursts (from DIV 17 to 28) in both male and female cultures (Fig. 5). Network burst duration was increased from DIV 21 to 28 after exposure to 100 µM chlorpyrifos in both sexes (Fig. 5), with a larger increase in females at DIV 28 (F (54, 1535) = 3.15). Burst duration was only affected in female cultures at DIV 28 (Fig. S6), but this effect was not sex-specific. The number of spikes per (network) burst and inter-burst interval were not affected by exposure to chlorpyrifos (Fig. S6). Exposure to 100 µM increased mean ISI within network bursts from DIV 17 to 28 in both sexes, with the increase being more profound in female cultures (Fig. 5; F (54, 1516) = 4.78). Synchronicity was decreased in female (from DIV 24 to 28) but not in male cultures (Fig. S6). This effect was not sex-specific.

**Fig. 6:**
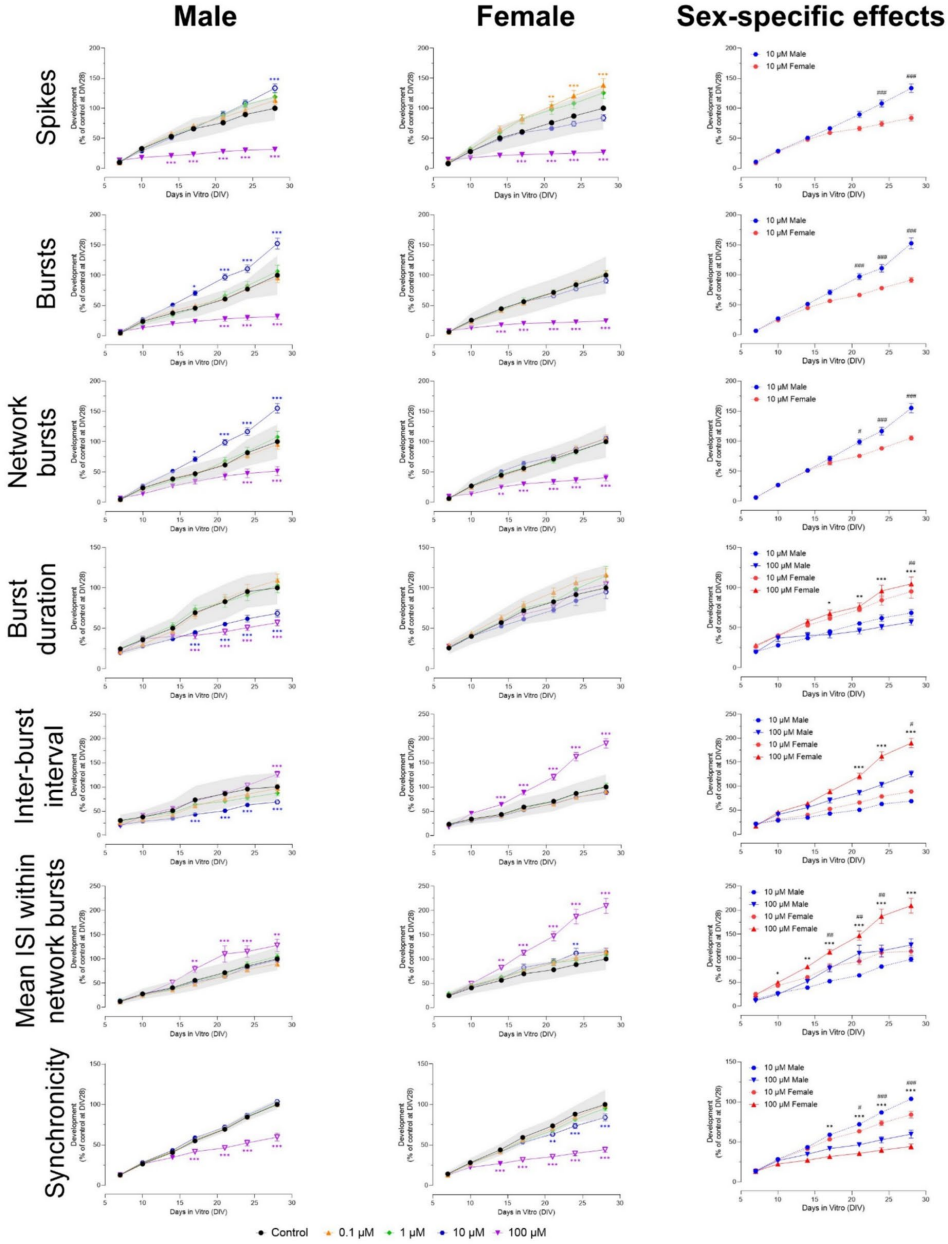
Exposure to chlorpyrifos-oxon (DIV 7–28) affects a subset of neuronal activity parameters in both male (left panel) and female (middle panel) cultures. The grey shaded area represents a benchmark response derived from the variation in DMSO control experiments. Data points display the average percentage compared to control (DMSO control at DIV 28 set to 100%) ± SEM from 13 to 24 individual wells (≥ 2 independent experiments per concentration). Difference from DMSO control (*p ≤ 0.05; **p ≤ 0.01; ***p ≤ 0.001). The color of asterisks indicates which concentration is significantly affected. Differences between sexes are depicted as open symbols. Exposure to chlorpyrifos-oxon evokes sex-specific effects at 10 and 100 µM (right panel). Sex-specific differences (*/#*p* ≤ 0.05; **/##*p* ≤ 0.01; ***/### *p* ≤ 0.001) are depicted with hashtags for 10 µM and asterisks for 100 µM

Exposure to 100 µM chlorpyrifos-oxon decreased the number of spikes in both sexes during the entire 21-day exposure, but only reached significance from DIV 14 to 28 (male cultures) and DIV 17–28 (female cultures; Fig. 6). Exposure to 0.1 µM (only in female cultures, from DIV 21 to 28) and 10 µM (only in male cultures at DIV 28) increased the number of spikes (Fig. 6). The effect seen after exposure to 10 µM was sex-specific (F (54, 1249) = 9.88). Exposure to 100 µM chlorpyrifos-oxon decreased the number of (network) bursts in both sexes, while exposure to 10 µM increased the number of (network) bursts from DIV 17 to 28 in male but not in female cultures (Figs. 6 and S7). This effect was sex-specific for both bursts (F (54, 1266) = 11.7) and network bursts (F (54, 1277) = 7.49). Exposure to ≥ 10 µM decreased burst duration (from DIV 17 to 28) in male, but not female cultures (Fig. 6; F (54, 1211) = 2.14). Network burst duration was also only decreased (from DIV 14–28) in male cultures (Fig. S8), but this effect was not sex-specific. The number of spikes per (network) burst decreased from DIV 14 to 28 in both sexes after exposure to 10 (only reaching significance in male cultures) and 100 µM (Fig. S8). Inter-burst interval decreased from DIV 17 to 28 after exposure to 10 µM in male cultures, reflecting the increase in the number of bursts. Exposure to 100 µM increased inter-burst interval in both male (only at DIV 28) and female (from DIV 14 to 28; Fig. 6) cultures. Both 10 and 100 µM evoked sex-specific effects on inter-burst interval (F (54, 1211) = 9.67). Mean ISI within network bursts increased after exposure to 10 µM (only in female cultures) and 100 µM, with the increase being more profound and earlier in female cultures (Fig. 6; F (54, 1171) = 6.07). Finally, synchronicity decreased after exposure to 10 µM (only in female cultures) and 100 µM, with a more profound and earlier decrease in female cultures (Fig. 6; F (54, 1247) = 18.6).

**Fig. 7:**
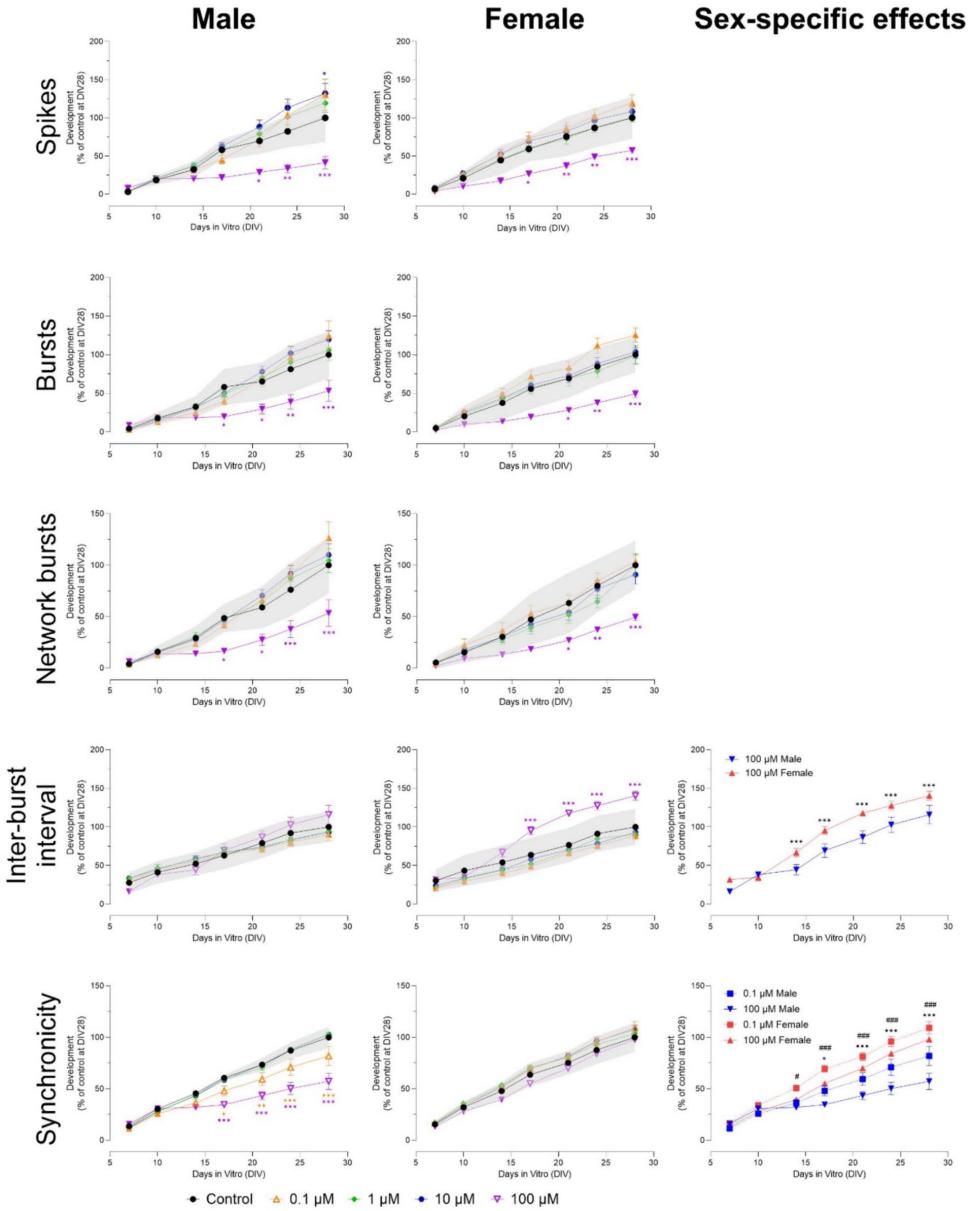
Exposure to TCP (DIV 7–28) affects a subset of neuronal activity parameters in both male (left panel) and female (middle panel) cultures. The grey shaded area represents a benchmark response derived from the variation in DMSO control experiments. Data points display the average percentage compared to control (DMSO control at DIV 28 set to 100%) ± SEM from 13 to 24 individual wells (≥ 2 independent experiments per concentration). Difference from DMSO control (* p ≤ 0.05; ** *p* ≤ 0.01; *** *p* ≤ 0.001). The color of asterisks indicates which concentration is significantly affected. Differences between sexes are depicted as open symbols. Exposure to TCP evokes sex-specific effects at 0.1 and 100 µM (right panel). Sex-specific differences (*/#*p* ≤ 0.05; **/##*p* ≤ 0.01; ***/###*p* ≤ 0.001) are depicted with hashtags for 0.1 µM and asterisks for 100 µM

Exposure to 100 µM TCP decreased the number of spikes and (network) bursts from DIV 17 to 28 in both sexes (Fig. 7). Network burst duration was not affected in both sexes, while exposure to 10 µM increased burst duration from DIV 24 to 28 in male but not in female cultures (Fig. S9). Moreover, in male cultures burst duration was increased at DIV 28 after exposure to 0.1 and 1 µM, but decreased after exposure to 100 µM. The number of spikes per (network) burst was decreased from DIV 14 to 28 after exposure to 100 µM TCP in male but not in female cultures (Fig. S9). The number of spikes per burst was not affected in female cultures. Exposure to 10 µM (at DIV 28) and 100 µM (from DIV 24 to 28) increased the number of spikes per network burst in females (Fig. S9). These effects were not sex-specific. Inter-burst interval increased from DIV 17–28 in female cultures after exposure to 100 µM, while no effects were found in male cultures (Fig. 7; F (54, 955) = 1.72). Mean ISI within network bursts increased in male cultures from DIV 21 to 28 after exposure to 100 µM, but decreased in female cultures from DIV 14 to 28 after exposure to 10 µM TCP (Fig. S9). These effects were not sex-specific. Finally, synchronicity decreased from DIV 17 to 28 in male cultures after exposure to 0.1 and 100 µM, but not in female cultures (Fig. 7; F (54, 983) = 2.63).

Concluding, exposure to ≥ 10 µM CPF caused a hyperexcitation in both male and female cultures after 1 to 2 weeks of exposure. Some small sex-specific effects were found on network burst duration and mean ISI within network bursts. Exposure to 100 µM CPO decreased neuronal development in both sexes after only 1 week of exposure. Interestingly, exposure to 10 µM CPO caused a clear sex-specific effect. In male cultures, a hyperexcitation was observed after 1–2 weeks of exposure, reflected by an increase in the number of spikes and (network) bursts. However, these (network) bursts were also shorter and had fewer spikes per (network) burst. No clear effects were found in female cultures after 10 µM exposure, suggesting that males are more susceptible to CPO exposure than females. Exposure to 100 µM TCP decreased neuronal development in both sexes after 2 weeks of exposure. Some small sex-specific effects were found on inter-burst interval. A stronger effect was found on synchronicity, which was more affected in male than in female cultures. The effects found here after exposure to chlorpyrifos, chlorpyrifos-oxon, and TCP are not due to cytotoxicity (Fig. S10).

### Pyrethroids

**Fig. 8:**
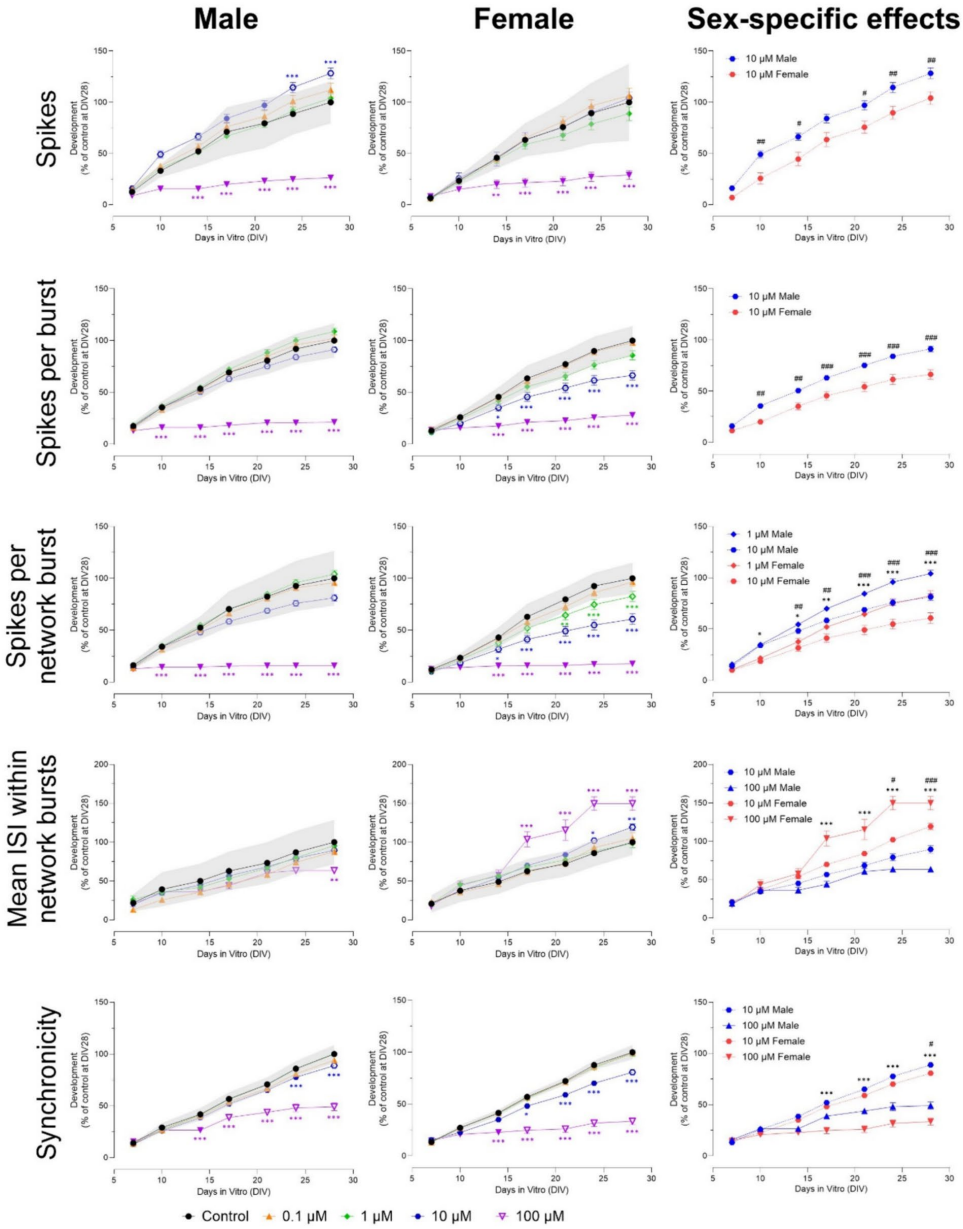
Exposure to permethrin (DIV 7–28) affects a subset of neuronal activity parameters in both male (left panel) and female (middle panel) cultures. The grey shaded area represents a benchmark response derived from the variation in DMSO control experiments. Data points display the average percentage compared to control (DMSO control at DIV 28 set to 100%) ± SEM from 14–23 individual wells (≥ 2 independent experiments per concentration). Difference from DMSO control (*p ≤ 0.05; **p ≤ 0.01; ***p ≤ 0.001). The color of asterisks indicates which concentration is significantly affected. Differences between sexes are depicted as open symbols. Exposure to permethrin evokes sex-specific effects at 1, 10 and 100 µM (right panel). Sex-specific differences (*/#/$*p* ≤ 0.05; **/##/$$*p* ≤ 0.01; ***/###/$$$ *p* ≤ 0.001) are depicted with dollar signs for 1 µM, hashtags for 10 µM and asterisks for 100 µM

Exposure to 100 µM permethrin decreased the number of spikes from DIV 14 to 28 in both sexes, while exposure to 10 µM increased the number of spikes in male, but not female cultures (Fig. 8). This effect was sex-specific (F (54, 1024) = 5.95). The number of bursts decreased in male cultures (DIV 24–28) and female cultures (DIV 17–28) after exposure to 100 µM permethrin, but increased after exposure to 10 µM in both male (DIV 24–28) and female cultures (only at DIV 28; Fig. S11). Exposure to 100 µM permethrin decreased the number of network bursts in male cultures at DIV 28. This effect was sex-specific (F (54, 1023) = 3.17). Exposure to 10 µM increased the number of network bursts from DIV 17 to 28 (male cultures) and DIV 21–28 (female cultures; Fig. S11). Burst duration decreased after exposure to 100 µM (male cultures) and ≥ 10 µM (female cultures; Fig. S11). A sex-specific effect was found at DIV 21 and 24 after exposure to 10 µM (F (54, 936) = 5.93). Network burst duration was affected at lower concentrations than burst duration. In male cultures, network burst duration was decreased after exposure to ≥ 10 µM (Fig. S11). In female cultures, the decrease in network burst duration occurred earlier (from DIV 21 to 28) and was more profound, with the decrease after exposure to 1 µM also reaching significance at DIV 28 (Fig. S11 and Fig. S12). The number of spikes per (network) burst decreased from DIV 10–28 in both sexes after exposure to 100 µM. In female cultures, this decrease was also observed after exposure to 10 µM (from DIV 14 to 28) and even after exposure to 1 µM (from DIV 21 to 28, only reaching significance in the number of spikes per network bursts; Fig. 8 and Fig. S12). The effects found at 1 and 10 µM were sex-specific for both number of spikes per burst (F (54, 994) = 15.3), and number of spikes per network burst (F (54, 995) = 13.6). Inter-burst interval decreased from DIV 24 to 28 after exposure to 10 and 100 µM in male but not in female cultures (Fig. S11; F (54, 936) = 1.38). Mean ISI within network bursts increased after exposure to 10 (DIV 24–28) and 100 µM (DIV 17 to 28) in female, but not in male cultures. In male cultures, mean ISI within network bursts decreased at DIV 28 (Fig. 8). This effect was sex-specific (F (54, 932) = 2.48). Finally, synchronicity decreased in both male and female cultures after exposure to ≥ 10 µM, with more profound effects found in female cultures (Fig. 8; F (54, 1001) = 28.2).

**Fig. 9:**
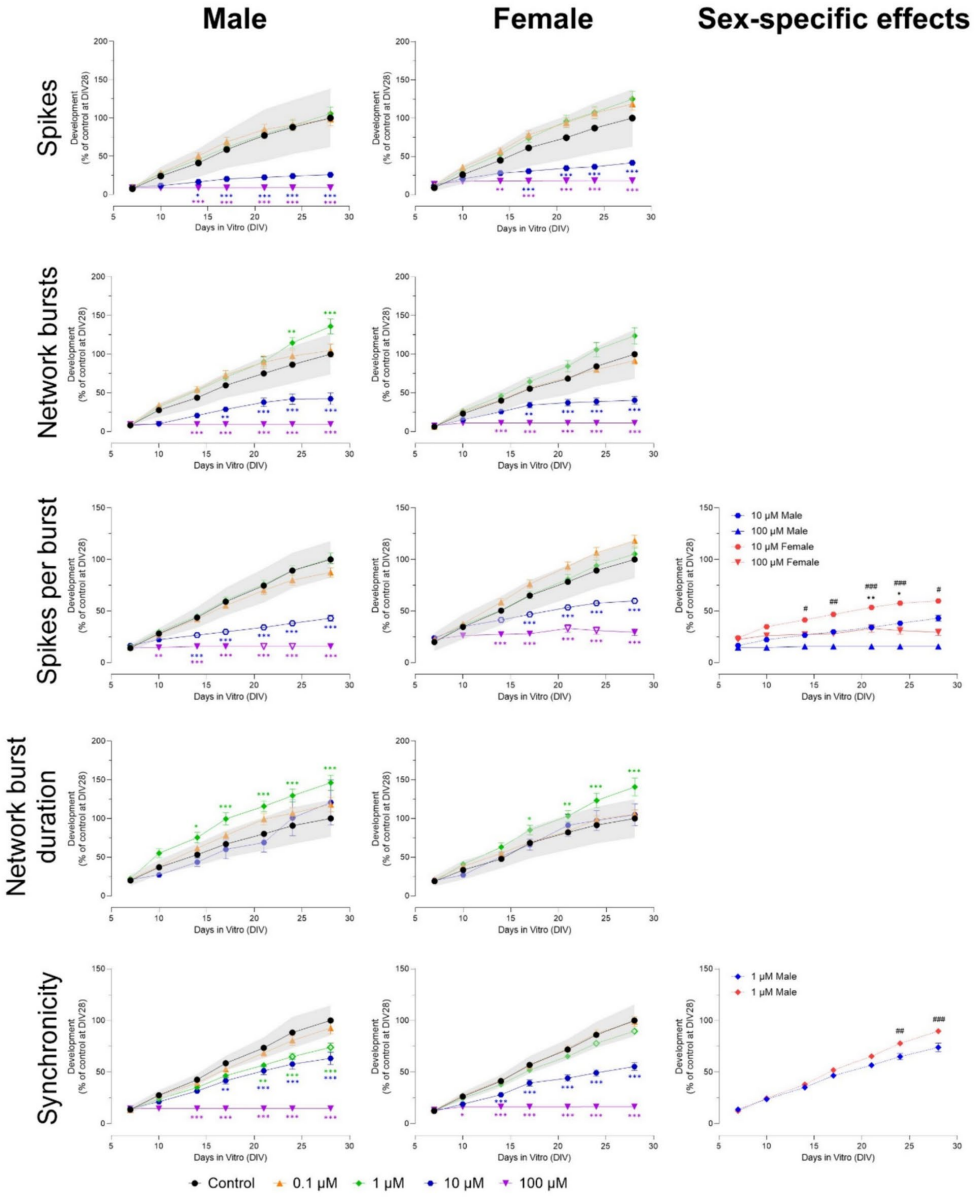
Exposure to alpha-cypermethrin (DIV 7–28) affects a subset of neuronal activity parameters in both male (left panel) and female (middle panel) cultures. The grey shaded area represents a benchmark response derived from the variation in DMSO control experiments. Data points display the average percentage compared to control (DMSO control at DIV 28 set to 100%) ± SEM from 17 to 30 individual wells (≥ 3 independent experiments per concentration). Difference from DMSO control (*p ≤ 0.05; **p ≤ 0.01; ***p ≤ 0.001). The color of asterisks indicates which concentration is significantly affected. Differences between sexes are depicted as open symbols. Exposure to alpha-cypermethrin evokes sex-specific effects at 1, 10 and 100 µM (right panel). Sex-specific differences Sex-specific differences (*/#/$*p* ≤ 0.05; **/##/$$*p* ≤ 0.01; ***/###/$$$*p* ≤ 0.001) are depicted with dollar signs for 1 µM, hashtags for 10 µM and asterisks for 100 µM

Exposure to ≥ 10 µM alpha-cypermethrin decreased the number of spikes and (network) bursts from DIV 14 to 28 (100 µM) and DIV 17 to 28 (10 µM) in both male and female cultures (Fig. 9 and Fig. S13). The number of network bursts increased from DIV 24–28 after exposure to 1 µM in both sexes, but only reached significance in male cultures (Fig. 9). Network burst duration increased after exposure to 1 µM alpha-cypermethrin in both male (from DIV 14 to 28) and female cultures (from DIV 17 to 28; Fig. 9). Burst duration was only affected in male cultures, with a decrease from DIV 14 to 28 after exposure to 10 µM (Fig. S13; F (42, 1164) = 2.51). The number of spikes per burst decreased from DIV 14 to 28 after exposure to ≥ 10 µM in both sexes (Fig. 9) but was more affected in males (F (54, 1544) = 18.2). The number of spikes per network burst also decreased from DIV 14 to 28 after exposure to ≥ 10 µM, but this effect was not sex-specific (Fig. S13). Inter-burst interval increased after exposure to 10 µM from DIV 10 to 28 (male cultures) and DIV 17–28 (female cultures; Fig. S13). A sex-specific effect was found at DIV 28, with females having a longer inter-burst interval (F (42, 1174) = 3.46). Mean ISI within network bursts increased from DIV 17 to 28 in both sexes after exposure to 1 and 10 µM (Fig. S13). Here, another sex-specific effect was found at DIV 28, with males having a higher mean ISI within network bursts (F (42, 1124) = 5.17). Because of the (almost) complete inhibition of activity at 100 µM, (network) burst duration, inter-burst interval and mean ISI within network bursts could not be determined for this concentration. Finally, synchronicity was decreased after exposure to 1 µM (from DIV 21 to 28, only in male cultures) and ≥ 10 µM (from DIV 14 to 28 in both sexes; Fig. 9). The effect found at 1 µM was sex-specific (F (54, 1536) = 28.4).

Exposure to 3-PBA had only limited effects on neuronal development (Fig. S14). Exposure to 0.1 µM 3-PBA increased the number of (network) bursts in male cultures at DIV 14 and 17, but not in female cultures or other time points. In female cultures, burst duration and spikes per burst were decreased after exposure to 100 µM (Fig. S14). Finally, exposure to 100 µM decreased synchronicity from DIV 24 to 28 in male cultures (Fig. S14). None of these effects were sex-specific.

Concluding, exposure to 100 µM permethrin decreased neuronal development in both male and female cultures after 1 week of exposure. Exposure to 10 µM permethrin resulted in hyperexcitation after 1–2 weeks after exposure, reflected by an increase in spikes and (network) bursts. In female cultures, these (network) bursts were also shorter with fewer spikes per (network) burst and the effects were seen at concentrations as low as 1 µM. These effects were sex-specific, suggesting that female cultures are more susceptible to permethrin exposure than male cultures. Exposure to ≥ 10 µM alpha-cypermethrin decreased neuronal development in both male and female cultures. Interestingly, 1 µM alpha-cypermethrin increased network burst duration (in both sexes) and decreased synchronicity (only in male cultures), indicating that disturbances in network development can also be found at lower exposure concentrations. Exposure to 3-PBA had only limited effects on neuronal development, with no clear concentration- or time-dependent patterns and no sex-specific effects. The effects found after exposure to permethrin and alpha-cypermethrin are not due to cytotoxicity (Fig. S15).

## Discussion

Our study aimed to determine the sex-specific effects of chronic insecticide exposure using MEA recordings, which enabled us to measure neuronal development over time in a non-invasive way. We found that chronic insecticide exposure can induce sex-specific effects in vitro. Moreover, changes in neuronal development after chronic insecticide exposure are also compound-specific, even between insecticides with similar modes of action. Comparing the present results with earlier, acute MEA data from our lab (van Melis et al. 2024) shows that acute and chronic exposure resulted in different, sometimes even opposite effects for some of the tested compounds, highlighting the value of both exposure paradigms. This is in line with another study comparing acute and chronic exposure (Martin et al. 2024), which suggests both assays are complementary to each other. To date, the availability of chronic MEA data on insecticide exposure is very limited, highlighting the novelty of our approach.

Spontaneous activity was observed in control cultures from DIV 4, after which activity slowly increased until DIV 10, indicating neuronal network development. After DIV 10, activity remained stable until DIV 28. The rat primary cortical networks used in this study are derived from PND 0–1 rat pups. These cells are already fully differentiated and have been isolated from an intact rat brain to single cells, which reform a network in vitro on our MEA plate. Since the cells are already fully differentiated from the start, the assay is not suitable to detect effects on early neurodevelopmental processes, such as neuronal migration and differentiation, but is rather aimed at detecting effects on network development (reformation) and maturation. At DIV 4, some treatment-unrelated sex-specific differences were found. However, activity at this time point was very low, making the quantification of these effects unreliable. Nevertheless, there could be (early) differences between cultures derived from males and females in neurophysiological correlates, such as BDNF signaling, synaptic density, and AMPA-or NMDA-receptor trafficking, that need to be examined more closely. Cell viability measurements demonstrated the absence of cell death at DIV 28 and neuronal activity could be increased by GABA antagonists (not shown), highlighting the viability of the neuronal cultures.

Carbamates and organophosphates primarily exert their neurotoxicity through the inhibition of acetylcholinesterase (AChE). However, we have previously shown that MEA recordings are not very sensitive to AChE inhibition (van Melis et al. 2023), most likely due to the low number of cholinergic neurons in our rat cortical model and thus very low ACh levels. Therefore, we conclude that the effects on neuronal network development after exposure to carbamates and organophosphates are most likely not due to AChE inhibition.

Exposure to 100 µM carbaryl induced sex-specific effects after 1 week of exposure, with an inhibition seen in male cultures and a hyperexcitation in female cultures. Earlier chronic MEA studies (Dingemans et al. 2016) tested concentrations of carbaryl up to 10 µM and used mixed cultures, which could explain the difference in results. Our results here are partly in line with earlier acute MEA results (see Table 1), in which exposure to > 40 µM decreased neuronal activity after acute exposure (Valdivia et al. 2014; van Melis et al. 2024). Since the effects in female and male cultures are opposite and only seen after 1 week of exposure, we expect that there may be multiple underlying mechanisms, including for example neurite outgrowth inhibition (J. Lee et al. 2022). Exposure to aldicarb and methomyl resulted in a hyperexcitation after 2 weeks of exposure that was more pronounced in female than in male cultures. These results are different from earlier acute MEA results (Table 1), in which both methomyl and aldicarb did not affect neuronal activity (van Melis et al. 2024). The mode of action underlying the excitation observed after chronic exposure to carbaryl (in females), aldicarb and methomyl is unknown and warrants further investigation. The sex-specific effects found after exposure to carbaryl and methomyl suggest that females might be more susceptible to the hyperactivity effects caused by carbamate exposure than males. This effect could possibly be related to hormonal pathways, such as the estrogen, androgen and progesterone receptor, which are known to differ between males and females (Cao and Patisaul 2013; Lana et al. 2023; Ravizza et al. 2002). As of yet, little is known about the developmental neurotoxicity of methomyl and aldicarb, highlighting the novelty of our results.

**Table 1:** List of compounds studied here with their acute neurotoxic effect on the MEA (acute MEA), developmental neurotoxic effect on the MEA (chronic MEA), and the in vitro lowest effect concentration (LOEC) found on the MEA in both assays

Compound	Acute MEA (LOEC)	Chronic MEA (LOEC)
Carbaryl	♂/♀Inhibition (100 µM)	♂ Inhibition (100 µM) ♀ Excitation (100 µM)
Aldicarb	♂/♀ No effect	♂/♀ Excitation (10 µM)
Methomyl	♂/♀ No effect	♂ Excitation (100 µM) ♀ Excitation (10 µM)
Chlorpyrifos	♂/♀ Inhibition (10 µM)	♂/♀ Excitation (10 µM)
Chlorpyrifos-oxon	♂/♀ Inhibition (1 µM)	♂ Excitation (10 µM) ♀ Inhibition (100 µM)
TCP	♂/♀ No effect	♂/♀ Inhibition (100 µM)
Permethrin	♂/♀ Excitation (10 µM)	♂/♀ Excitation (10 µM)
α-Cypermethrin	♂/♀ Inhibition (1 µM)	♂/♀ Inhibition (10 µM)
3-PBA	♂/♀ No effect	♂/♀ No effect

Effects and LOEC for acute MEA are based on van Melis et al. 2024

Exposure to ≥ 10 µM chlorpyrifos (CPF) caused a hyperexcitation in both sexes after 10 days of exposure. These results are in contrast to earlier MEA studies that found that after both acute and chronic exposure ≥ 10 µM CPF decreased neuronal activity (see Table 1; Dingemans et al. 2016; Valdivia et al. 2014; van Melis et al. 2023, 2024). Moreover, chronic exposure to organophosphates usually has effects associated with an inhibition in neuronal development, such as inhibition of axonal transport (Naughton and Terry 2018; Richardson et al. 2019) and decreased neurite outgrowth (Das and Barone 1999). The increase in neuronal activity seen here after CPF exposure could be due to adaptive mechanisms such as changes in the expression of ion channels and neurotransmitter receptors. For instance, CPF exposure can also increase brain-derived neurotrophic factor (BDNF) during development (Betancourt et al. 2007), which can in turn modulate synaptic activity (Lu 2003). Also, CPF upregulates expression of NDMA receptor subunits NR2A and NR2B (Gultekin et al. 2007) and the effects of chlorpyrifos neurotoxicity were attenuated by antagonists of the NMDA or AMPA receptor (Rush et al. 2010).

Notably, there was a clear difference in effect between CPF and chlorpyrifos-oxon (CPO). While exposure to ≥ 10 µM CPF induced a hyperexcitation, exposure to 100 µM CPO strongly inhibited neuronal development. Interestingly, exposure to 10 µM CPO induced sex-specific effects, with a clear hyperexcitation in male cultures after 10 days of exposure, but no clear effects in female cultures. The clear inhibition in neuronal development is in line with earlier MEA results after acute exposure (see Table 1; Valdivia et al. 2014; van Melis et al. 2024)) and might be attributed to a reduced depolarization-evoked Ca^2+^ influx (van Melis et al. 2023), but can also be caused by disruption of axonal transport and changes in neurite outgrowth. However, the sex-specific hyperexcitation after exposure to 10 µM CPO has not been seen before. This effect might be related to alterations in BDNF signaling, since studies have shown that male BNDF knock out mice show hyperactivity, while females BDNF knock out mice show normal locomotor activity (Monteggia et al. 2007; Rios et al. 2001). Surprisingly, exposure to 100 µM of the presumably inactive organophosphate metabolite TCP inhibited neuronal development after 2 weeks of exposure. In earlier acute MEA studies, TCP did not affect neuronal activity (Table 1; van Melis et al. 2024). While important from a mechanistic point of view, the effects found at 100 µM are at a concentration that far exceeds human internal exposure levels, thus limiting the toxicological relevance of these results.

Pyrethroids exert their neurotoxicity primarily by modifying the kinetics of voltage-gated sodium channels, resulting in the prolonged opening of these channels and subsequent alterations in membrane excitability and neuronal activity (Narahashi 1996; Shafer et al. 2005). Based on clinical symptoms and structure, they can be divided into type I and type II pyrethroids. Type I compounds generally prolong action potentials, resulting in repetitive firing. Type II pyrethroids also cause prolonged repetitive firing, but also the cessation of action potential generation by a depolarizing block (Narahashi 1996). Exposure to the type I pyrethroid permethrin resulted in a hyperexcitation at 10 µM and a decrease in neuronal development at 100 µM in both sexes. In female but not in male cultures, exposure to 1 and 10 µM also changed activity patterns, with shorter (network) bursts and fewer spikes per (network) burst. The hyperexcitation seen after exposure to 10 µM parallels MEA results after acute exposure, in which hyperactivity was seen after 10 and 30 µM (see Table 1; van Melis et al. 2024) and might be caused by the repetitive firing associated with type I pyrethroids. Similar to earlier results from acute and chronic MEA (see Table 1; Dingemans et al. 2016; van Melis et al. 2024), exposure to ≥ 10 µM alpha-cypermethrin decreased neuronal development in both sexes. This is in concordance with AOP 442 (https://aopwiki.org/aops/442), which states that disruption of sodium channel kinetics and action potential generations during development can lead to alteration of neurotransmission and eventually a decline in cognitive function. Alpha-cypermethrin also decreased motor activity and impaired learning in rats (Gómez-Giménez et al. 2018), and reduced neuronal proliferation, maturation, and differentiation in mice (Guo et al. 2018). Magby and Richardson (2017) found that developmental exposure to another pyrethroid, deltamethrin, down-regulated voltage-gated sodium channels in mice. As these changes can lead to persistent changes in neuronal function, it might explain the effects found here. Another possible explanation is the depolarization-dependent block of action potentials caused by type II pyrethroids. Similar to acute exposure, chronic exposure to the presumably inactive pyrethroid metabolite 3-PBA had no clear effects on neuronal development (Table 1).

Interestingly, exposure to 1 µM permethrin (only female cultures) and 1 µM alpha-cypermethrin (both sexes) also affected some neuronal activity parameters, indicating that disturbances in network development can also be found at lower exposure concentrations. Concentrations of 1 µM are close to reported human internal exposure levels. In blood from pregnant women in Taiwan, permethrin and cypermethrin levels could reach as high as 165 and 153 µg/mL respectively, which relates to an estimated internal concentration of 0.42 µM for permethrin and 0.37 µM for cypermethrin (Simaremare et al. 2020). Levels were even higher in cord blood from newborns in China, reaching 470 ng/mL (1.2 µM) for permethrin and 390 ng/mL (0.94 µM) for cypermethrin (Silver et al. 2015). The actual concentrations in the brain could differ from these values due to metabolism or barrier effects. It is important to note that, while some of the previously observed sex-specific effects in in vivo studies can be due to sex-specific differences in metabolism or distribution, the chronic sex-specific effects found in this in vitro study are unlikely to be related to sex-specific ADME differences. Hence, it seems that there are subtle differences in neuronal characteristics between female and male cortical cultures, such as possible differences in the amount of specific neurotransmitter receptors or ion channels (Brandt et al. 2020; Chang et al. 2007; Knouse et al. 2022, 2023), that remain undetected until a chemical challenge.

The parameters affected by exposure to 1 µM permethrin and alpha-cypermethrin are not commonly reported parameters of neuronal activity (such as mean spike/burst rate) that MEA research usually focuses on. In this study, we reported the results of the ten most important MEA parameters, based on an earlier PCA (van Melis et al. 2024). This expands our knowledge of neuronal activity patterns after chronic insecticide exposure and enables us to detect human-relevant effects that would otherwise have been missed.

The current study shows effects after chronic exposure starting from DIV 7. However, at DIV 7 particular stages in neuronal development may already have passed. It may be possible that exposure during an earlier phase of network formation evokes different or more potent effects than those seen in the current chronic exposure paradigm. Furthermore, a lot of neurotoxic compounds are also endocrine-disrupting chemicals (EDCs). Since EDCs are known to induce sex-specific effects, it would be of interest to see if sex-specific developmental neurotoxicity is endocrine-mediated.

To conclude, our study shows that chronic insecticide exposure has different effects on neuronal development. Some of these effects are sex-specific. To our knowledge, this is the first time that sex-specific in vitro effects on neuronal activity after chronic exposure to insecticides have been investigated. It also demonstrates that the chronic MEA assay can be used to test sex-specific developmental neurotoxic effects, which may reduce the need for in vivo studies that are costly and require a large number of animals. Some of the effects found here are at concentrations close to human internal exposure levels, highlighting the need to lower the exposure of the general population to insecticides.

## Supplementary Information

Below is the link to the electronic supplementary material.

Supplementary file1 (DOCX 3974 KB)

## Data Availability

Data will be made available on request.
