# Supplementary material for: Effects of chronic insecticide exposure on neuronal network development in vitro in rat cortical cultures
Source: Arch Toxicol. 2024 Aug 20;98(11):3837–57. doi: 10.1007/s00204-024-03840-0 (PMC11489184; doi:10.1007/s00204-024-03840-0)
Supplement: Supplementary file 1 — Supplementary file1 (DOCX 3974 KB) [file 204_2024_3840_MOESM1_ESM.docx]

**Supplementary data**

**Effects of chronic insecticide exposure on neuronal network development in vitro in rat cortical cultures**

Lennart V.J. van Melis, Anneloes M. Peerdeman, Celia Arenas González, Regina G.D.M. van Kleef, J. Pepijn Wopken, Remco H.S. Westerink*

**Author affiliations**

Neurotoxicology Research Group, Division of Toxicology, Institute for Risk Assessment Sciences (IRAS), Faculty of Veterinary Medicine, Utrecht University, P.O. Box 80.177, NL-3508 TD Utrecht, The Netherlands

^*^ Correspondence and reprint requests to R.H.S. Westerink, Neurotoxicology Research Group, Division of Toxicology, Institute for Risk Assessment Sciences (IRAS), Faculty of Veterinary Medicine, Utrecht University, P.O. Box 80.177, NL-3508 TD Utrecht, The Netherlands; E-mail: R.Westerink@uu.nl

**Table S1**. Set of the ten most important micro-electrode array (MEA) parameters.

| Category | Metrics Parameters | Description |
| --- | --- | --- |
| Spike parameters | Number of spikes | Total number of spikes over the duration of the analysis |
|  | | |
| Burst  parameters | Number of bursts | Total number of bursts over the duration of the analysis |
|  | Burst duration | Average time from the first spike in a burst till the last spike (s). Longer bursts indicate more excitation as it takes longer to shut down a burst |
|  | Number of spikes per burst | Average number of spikes occurring in a burst |
|  | Inter-burst interval (IBI) | Time between the last spike of a burst and the first spike of a subsequent burst (s) |
|  | | |
| Network burst  parameters | Number of network bursts | Total number of network bursts over the duration of the analysis |
|  | Network burst duration | Average time from the first spike till the last spike in a network burst (s). Longer bursts indicate more excitation as it takes longer to shut down a burst |
|  | Number of spikes per network burst | Average number of spikes occurring in a network burst |
|  | Mean ISI within network burst | Average of the mean ISIs within a network burst (s) |
|  | | |
| Synchronicity parameters | Area under cross-correlation | Area under inter-electrode cross-correlation. The higher the value, the greater the synchronicity of the network |

**Figure S1.** Raster plots showing neuronal activity on DIV 28 in a single representative well after 21 days exposure to DMSO (left) and 100 µM carbaryl (right) in both males (A) and females (B). Each horizontal line represents activity on a single electrode, with a total of 16 electrodes per well. Spikes are represented as a single vertical line, bursts as a black box (spike train) and network bursts are shown in purple boxes spanning multiple electrodes. Synchronicity and the intensity of the (network) bursts is depicted in the cumulative black trace on top of the raster plot.

**A**


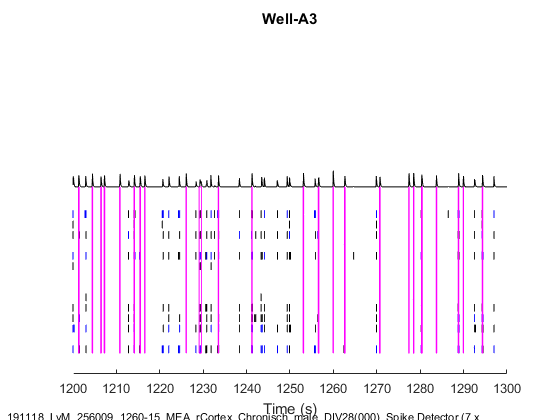

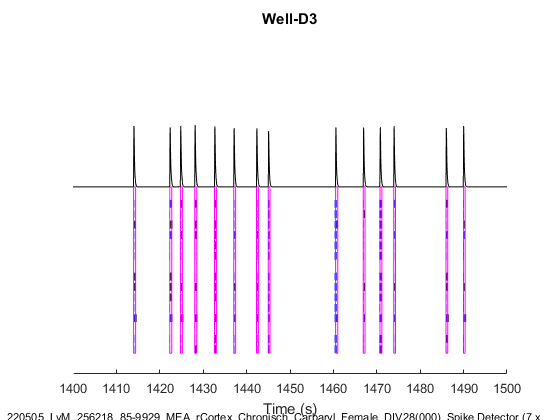

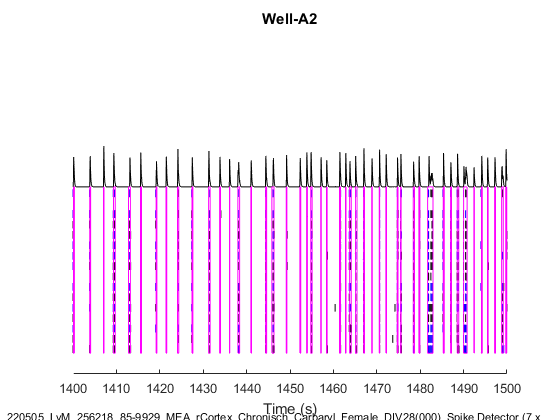

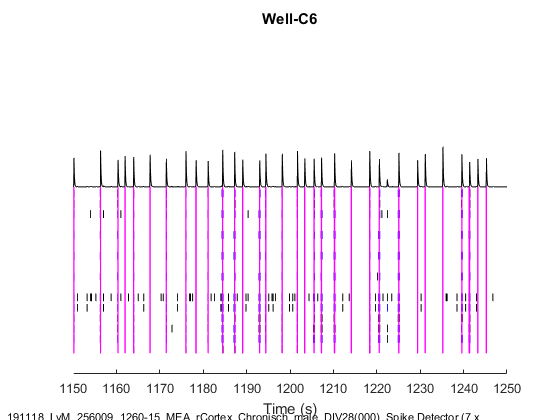


**B**


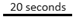

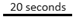

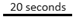

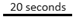


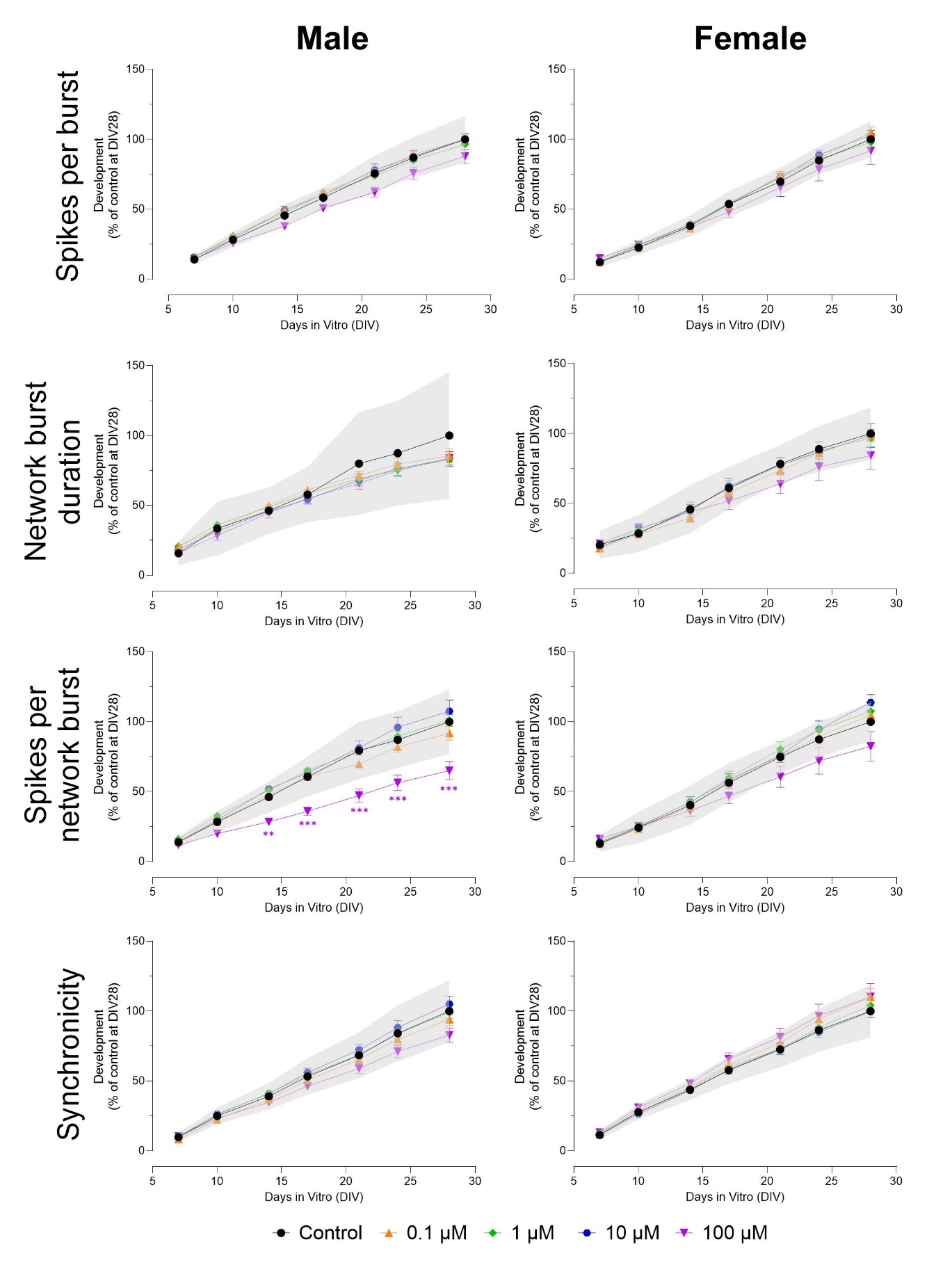
**Figure S2**. Effects of exposure to carbaryl (DIV 7-28) on a subset of neuronal activity parameters in both male (left) and female (right) cultures. The grey shaded area represents a benchmark response derived from the variation in DMSO control experiments. Data points display average percentage compared to control (DMSO control at DIV 28 set to 100%) ± SEM from 12-16 individual wells (≥ 2 independent experiments per concentration). Difference from DMSO control (* *p*≤0.05; ** *p*≤0.01; *** *p*≤0.001). Color of asterisks indicates which concentration is significantly affected.


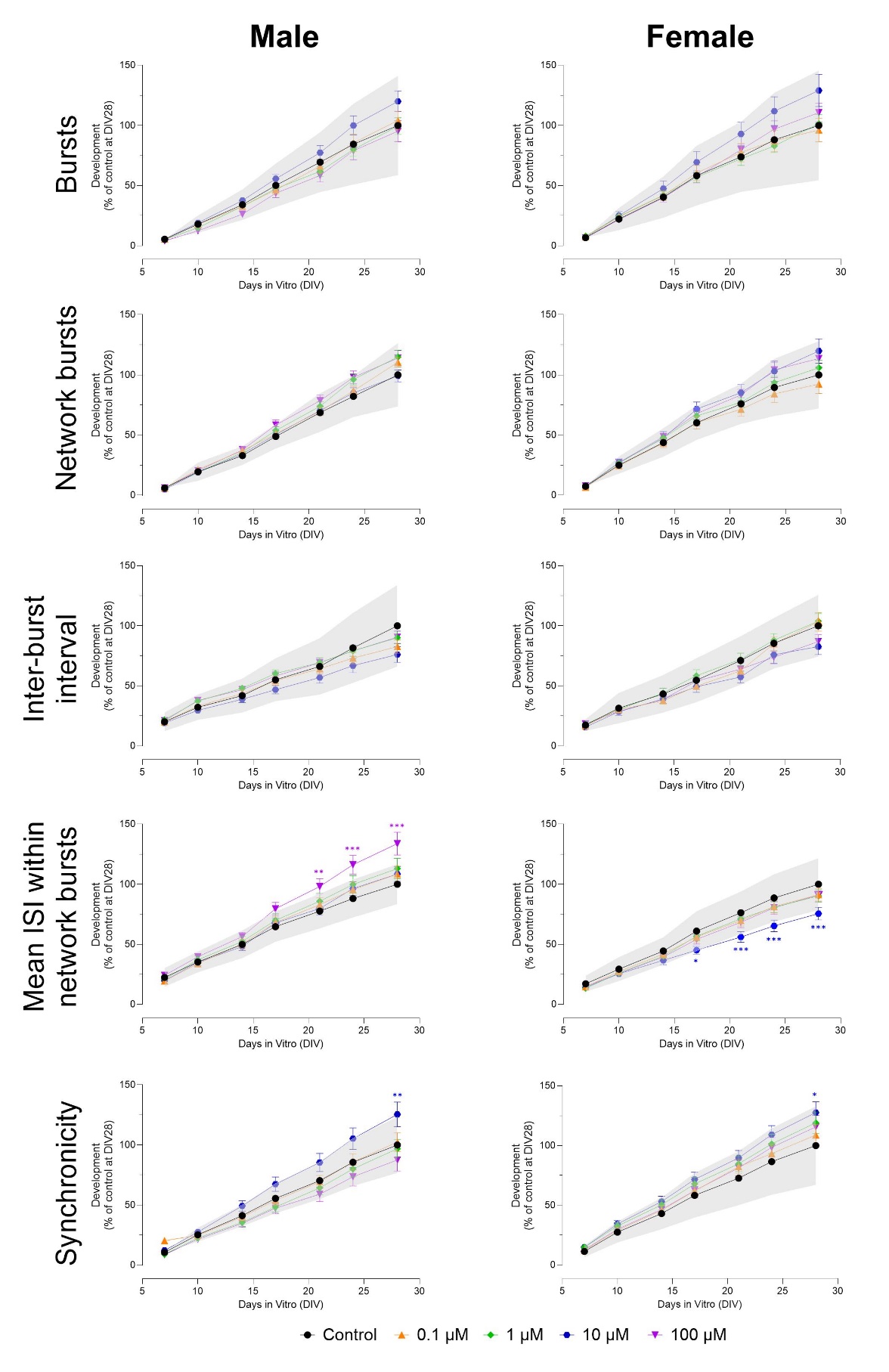
**Figure S3**. Effects of exposure to aldicarb (DIV 7-28) on a subset of neuronal activity parameters in both male (left) and female (right) cultures later in development. The grey shaded area represents a benchmark response derived from the variation in DMSO control experiments. Data points display average percentage compared to control (DMSO control at DIV 28 set to 100%) ± SEM from 13-22 individual wells (≥ 3 independent experiments per concentration). Difference from DMSO control (* *p*≤0.05; ** *p*≤0.01; *** *p*≤0.001). Color of asterisks indicates which concentration is significantly affected.


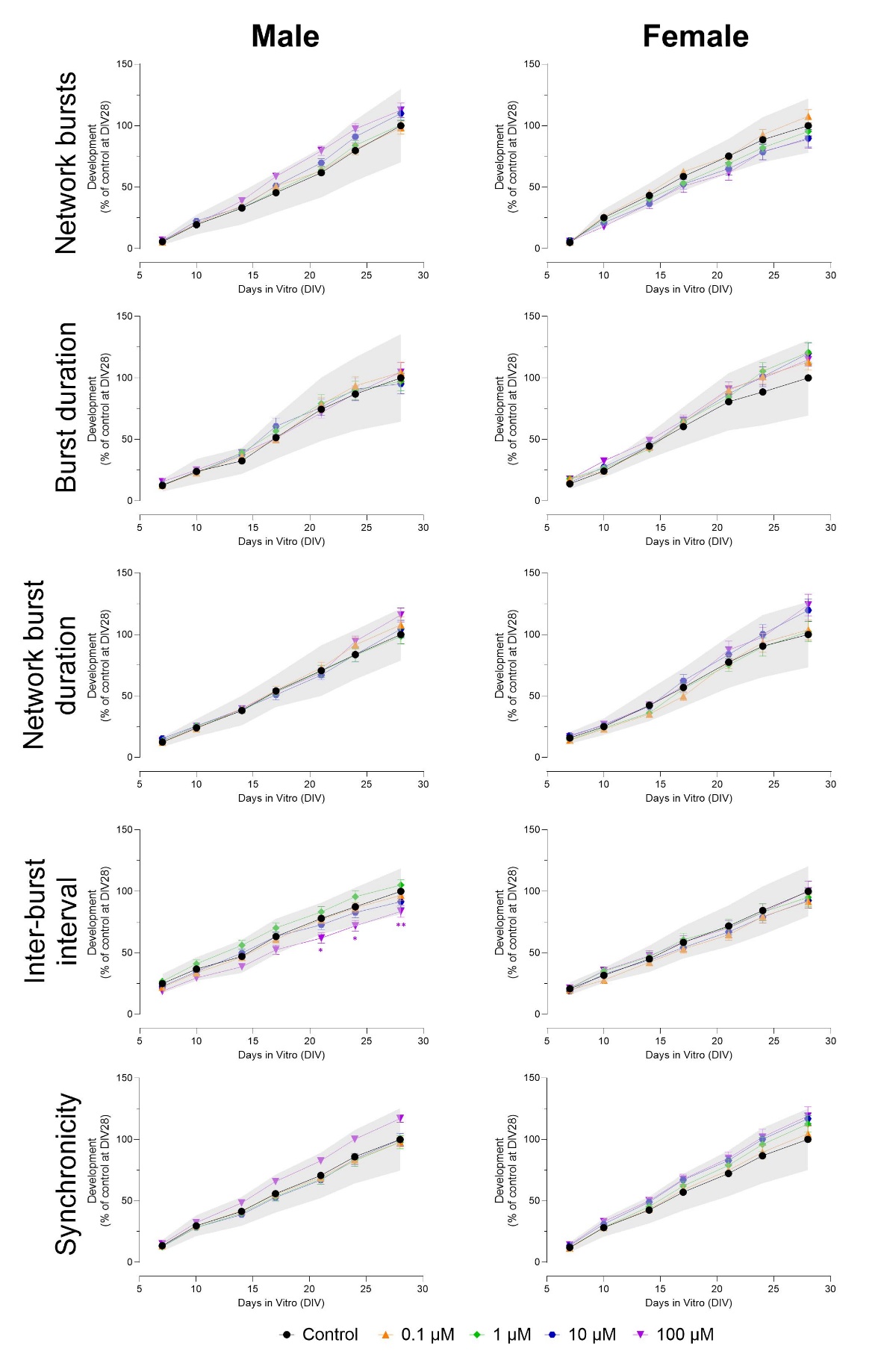
**Figure S4**. Effects of exposure to methomyl (DIV 7-28) on a subset of neuronal activity parameters in both male (left) and female (right) cultures. The grey shaded area represents a benchmark response derived from the variation in DMSO control experiments. Data points display average percentage compared to control (DMSO control at DIV 28 set to 100%) ± SEM from 16-22 individual wells (≥ 3 independent experiments per concentration). Difference from DMSO control (* *p*≤0.05; ** *p*≤0.01; *** *p*≤0.001). Color of asterisks indicates which concentration is significantly affected.

**
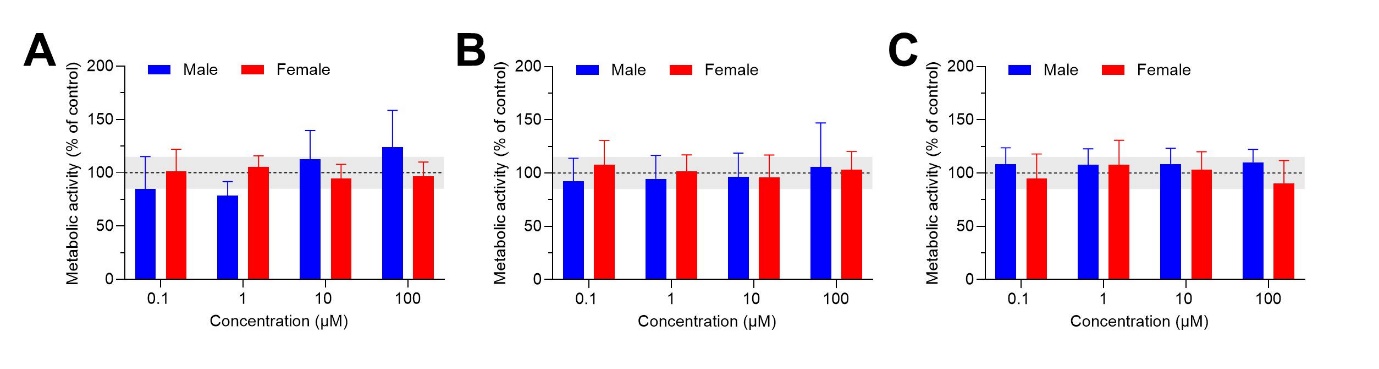
Figure S5.** Exposure (DIV 7-28) to 0.1-100 µM carbaryl (A), aldicarb (B), and methomyl (C) does not lead to changes in cell viability. The grey shaded area represents a benchmark response of 15%, which is derived from the average variation in DMSO control experiments. Bars represent the average percentage viability (± SD; ≥ 11 wells from ≥ 2 independent experiments) compared to control.


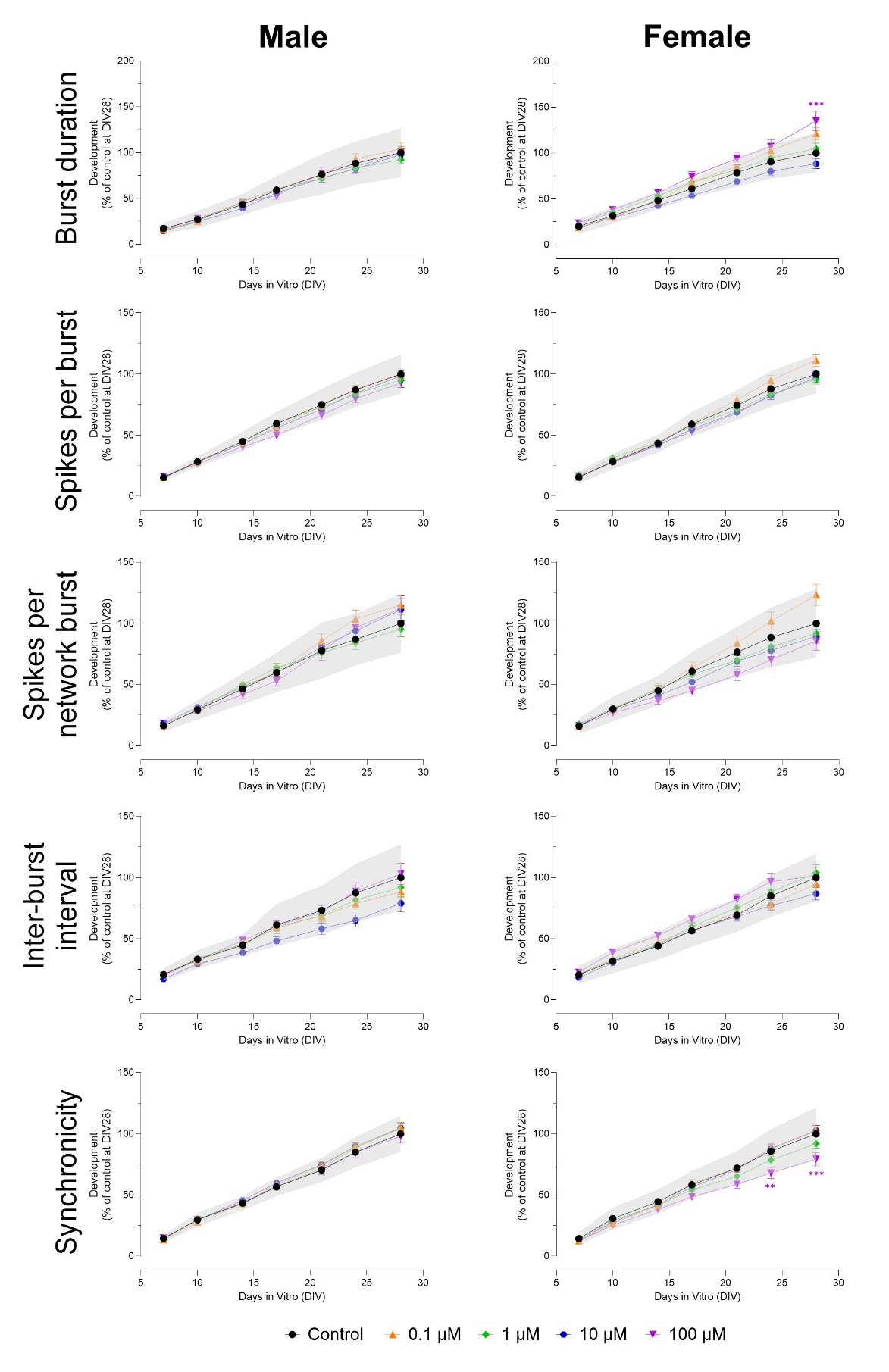
**Figure S6**. Effects of exposure to chlorpyrifos (DIV 7-28) on a subset of neuronal activity parameters in both male (left) and female (right) cultures. The grey shaded area represents a benchmark response derived from the variation in DMSO control experiments. Data points display average percentage compared to control (DMSO control at DIV 28 set to 100%) ± SEM from 20-32 individual wells (≥ 5 independent experiments per concentration). Difference from DMSO control (* *p*≤0.05; ** *p*≤0.01; *** *p*≤0.001). Color of asterisks indicates which concentration is significantly affected.

**Figure S7.** Raster plots showing neuronal activity on DIV 28 in a single representative well after 21 days exposure to DMSO (left) and 10 µM chlorpyrifos-oxon (right) in both males (A) and females (B). Each horizontal line represents activity on a single electrode, with a total of 16 electrodes per well. Spikes are represented as a single vertical line, bursts as a black box (spike train) and network bursts are shown in purple boxes spanning multiple electrodes. Synchronicity and the intensity of the (network) bursts is depicted in the cumulative black trace on top of the raster plot.

**B**


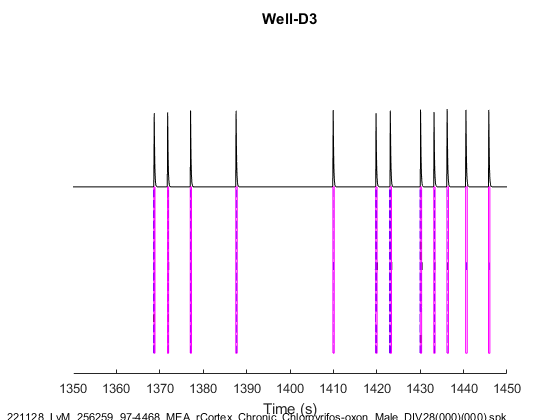

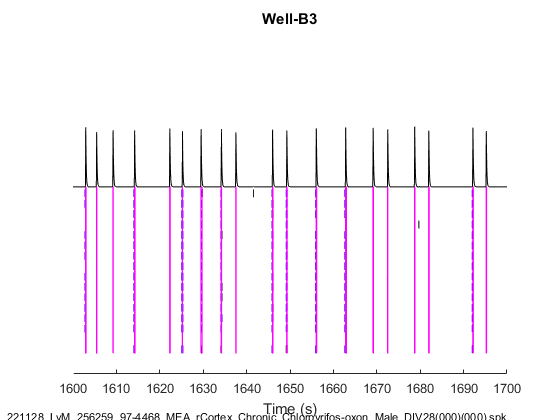

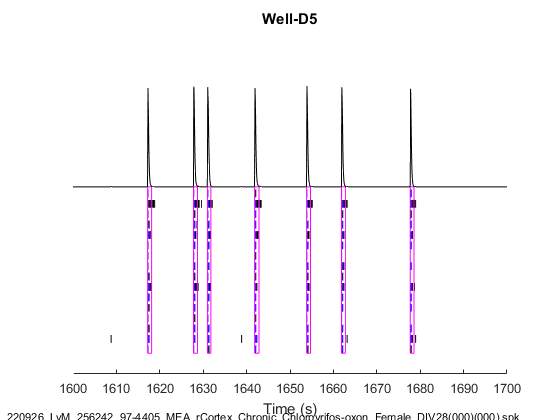

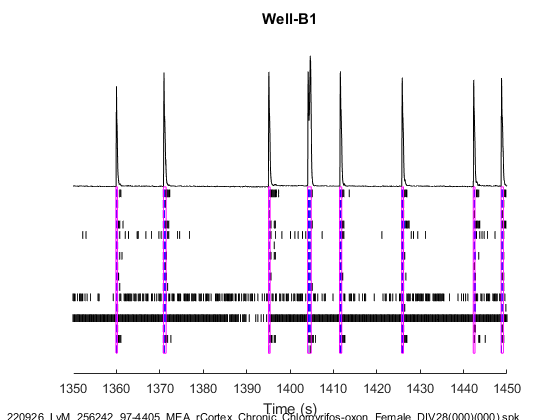


**A**


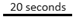

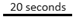

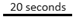

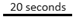


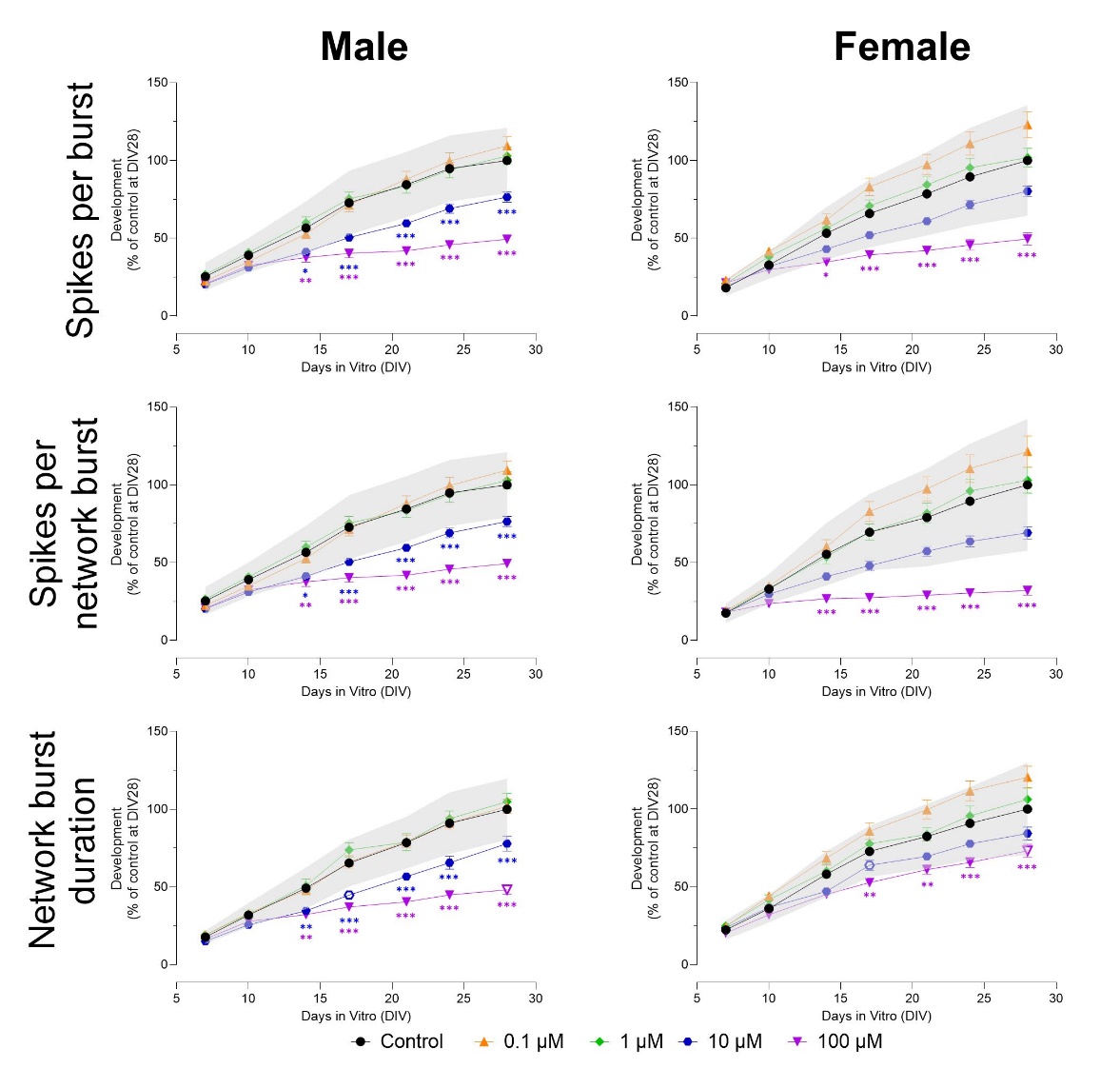
**Figure S8**. Effects of exposure to chlorpyrifos-oxon (DIV 7-28) on a subset of neuronal activity parameters in both male (left) and female (right) cultures. The grey shaded area represents a benchmark response derived from the variation in DMSO control experiments. Data points display average percentage compared to control (DMSO control at DIV 28 set to 100%) ± SEM from 12-24 individual wells (≥ 2 independent experiments per concentration). Difference from DMSO control (* *p*≤0.05; ** *p*≤0.01; *** *p*≤0.001). Color of asterisks indicates which concentration is significantly affected.


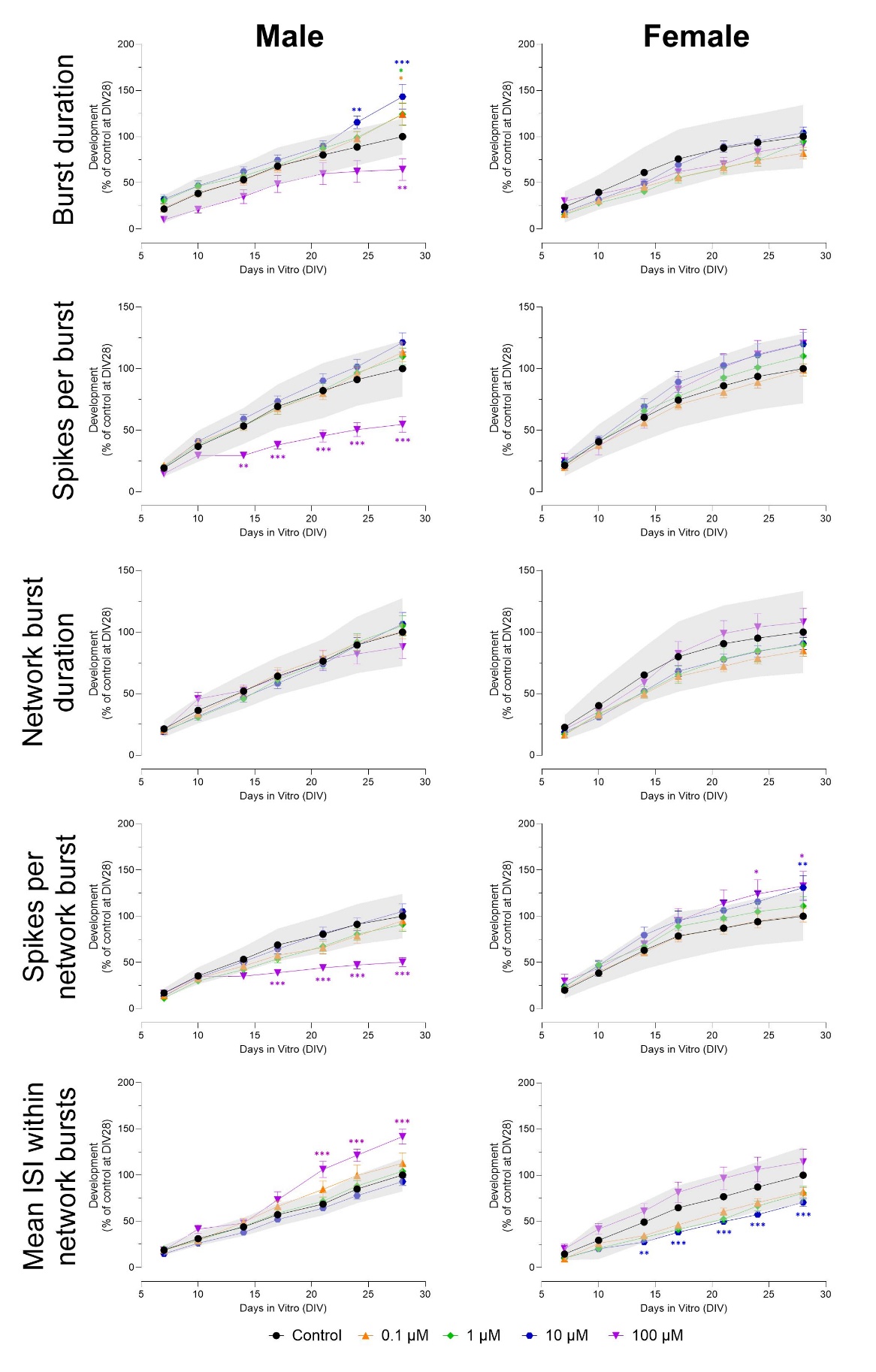
 **Figure S9**. Effects of exposure to 3,5,6-trichloropyridinol (TCP; DIV 7-28) on a subset of neuronal activity parameters in both male (left) and female (right) cultures. The grey shaded area represents a benchmark response derived from the variation in DMSO control experiments. Data points display average percentage compared to control (DMSO control at DIV 28 set to 100%) ± SEM from 13-24 individual wells (≥ 3 independent experiments per concentration). Difference from DMSO control (* *p*≤0.05; ** *p*≤0.01; *** *p*≤0.001). Color of asterisks indicates which concentration is significantly affected.


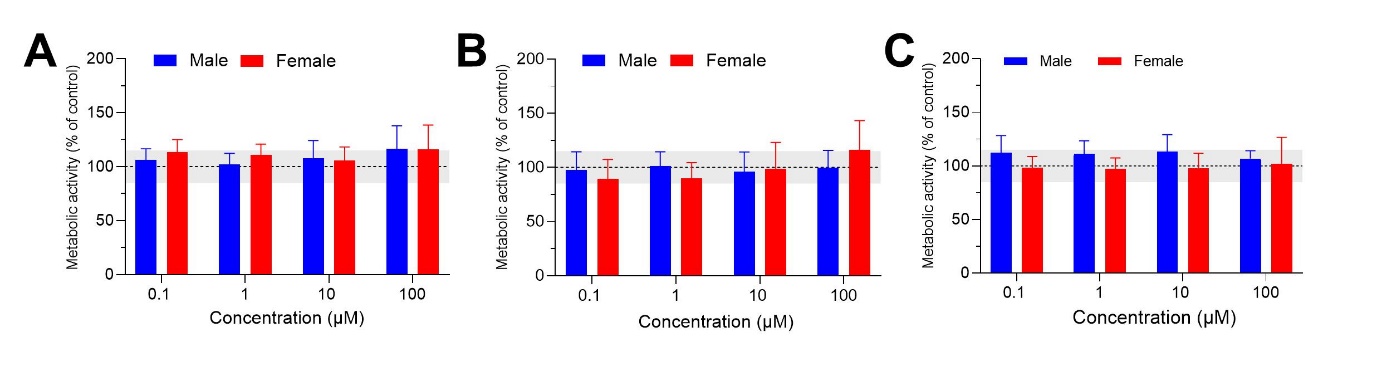
**Figure S10.** Exposure (DIV 7-28) to 0.1-100 µM chlorpyrifos (A), chlorpyrifos-oxon (B), and TCP (C) does not lead to changes in cell viability. The grey shaded area represents a benchmark response of 15%, which is derived from the average variation in DMSO control experiments. Bars represent the average percentage viability (± SD; ≥ 14 wells from ≥ 2 independent experiments) compared to control.


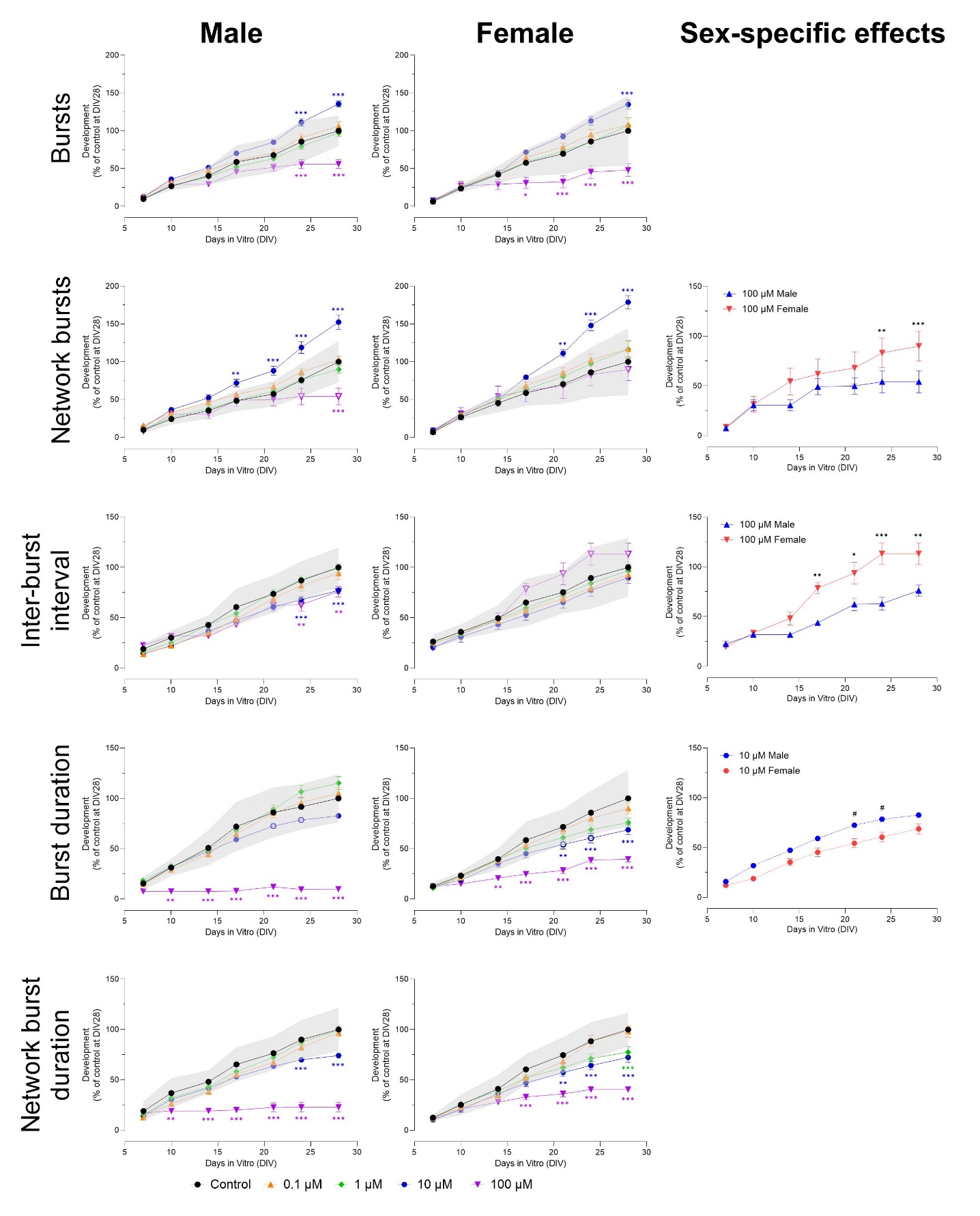
**Figure S11**. Effects of exposure to permethrin (DIV 7-28) on a subset of neuronal activity parameters in both male (left) and female (middle) cultures. The grey shaded area represents a benchmark response derived from the variation in DMSO control experiments. Data points display average percentage compared to control (DMSO control at DIV 28 set to 100%) ± SEM from 13-24 individual wells (≥ 2 independent experiments per concentration). Difference from DMSO control (* *p*≤0.05; ** *p*≤0.01; *** *p*≤0.001). Color of asterisks indicates which concentration is significantly affected. Differences between sexes (*p*≤0.05) are depicted as open symbols. Exposure to permethrin evokes sex-specific effects at 10 and 100 µM (right panel). Sex-specific differences (*/# *p*≤0.05; **/## *p*≤0.01; ***/### *p*≤0.001) are depicted with hashtags for 10 µM and asterisks for 100 µM.

**Figure S12.** Raster plots showing neuronal activity on DIV 28 in a single representative well after 21 days exposure to DMSO (left) and 1 µM permethrin (right) in both males (A) and females (B). Each horizontal line represents activity on a single electrode, with a total of 16 electrodes per well. Spikes are represented as a single vertical line, bursts as a black box (spike train) and network bursts are shown in purple boxes spanning multiple electrodes. Synchronicity and the intensity of the (network) bursts is depicted in the cumulative black trace on top of the raster plot.


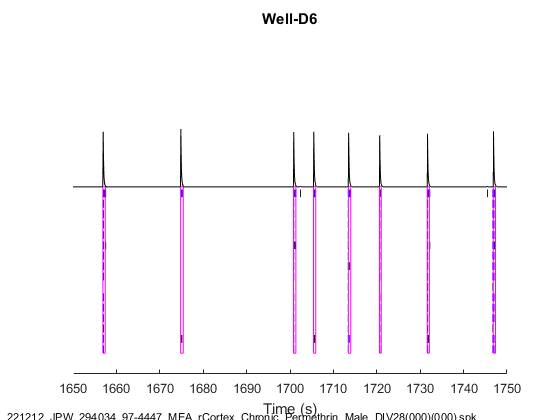

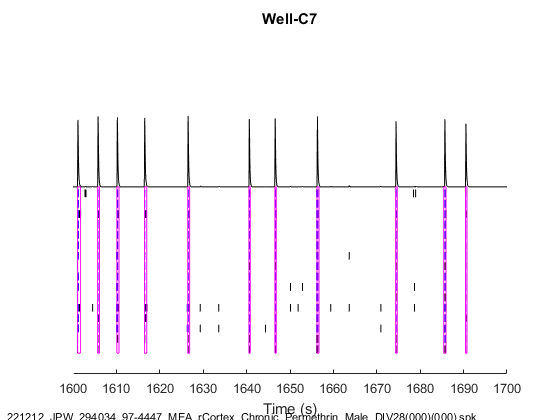

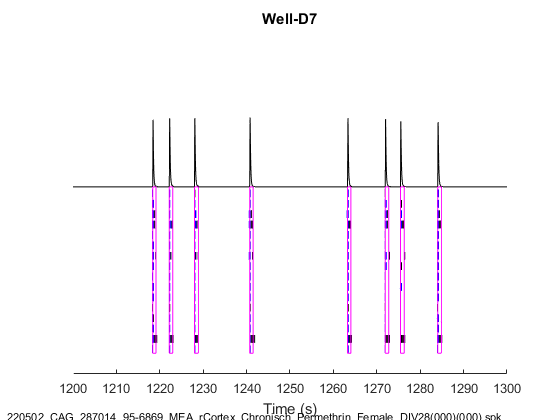

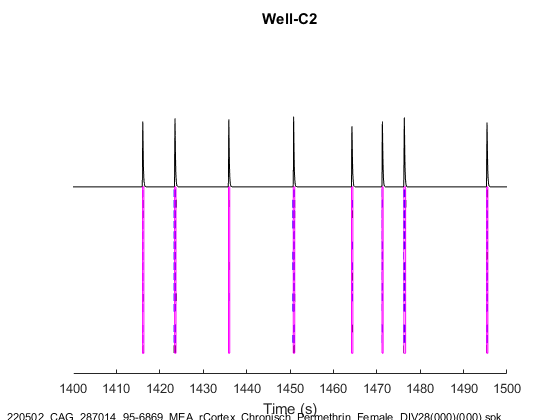


**A**

**B**


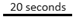

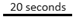

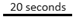

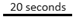


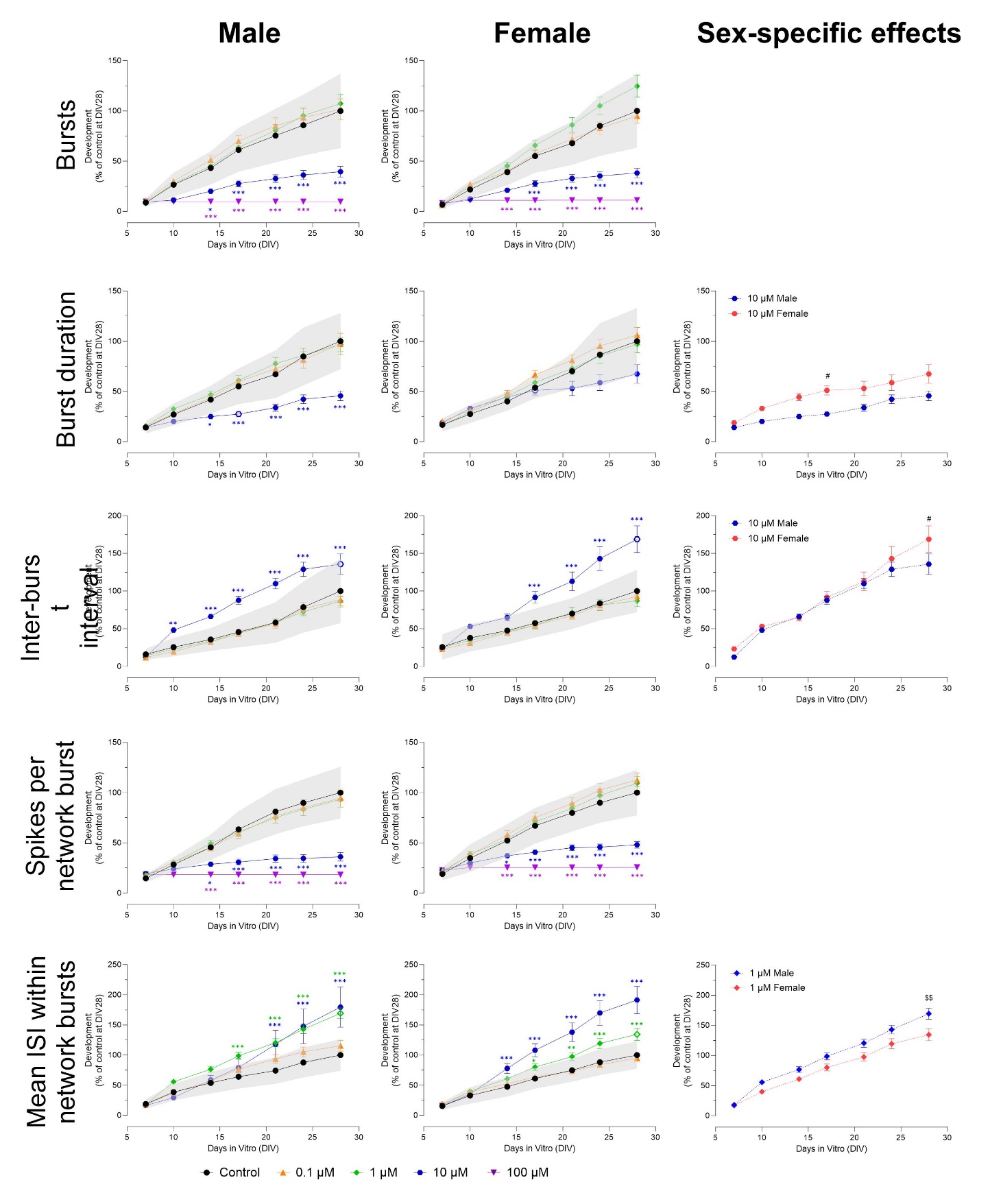
**Figure S13**. Effects of exposure to alpha-cypermethrin (DIV 7-28) on a subset of neuronal activity parameters in both male (left panel) and female (middle panel) cultures. The grey shaded area represents a benchmark response derived from the variation in DMSO control experiments. Data points display average percentage compared to control (DMSO control at DIV 28 set to 100%) ± SEM from 18-30 individual wells (≥ 3 independent experiments per concentration). Difference from DMSO control (* *p*≤0.05; ** *p*≤0.01; *** *p*≤0.001). Color of asterisks indicates which concentration is significantly affected. Differences between sexes (*p*≤0.05) are depicted as open symbols. Exposure to alpha-cypermethrin evokes sex-specific effects at 1 and 10 µM (right panel). Sex-specific differences (# *p*≤0.05; ## *p*≤0.01; ### *p*≤0.001) are depicted with hashtags.


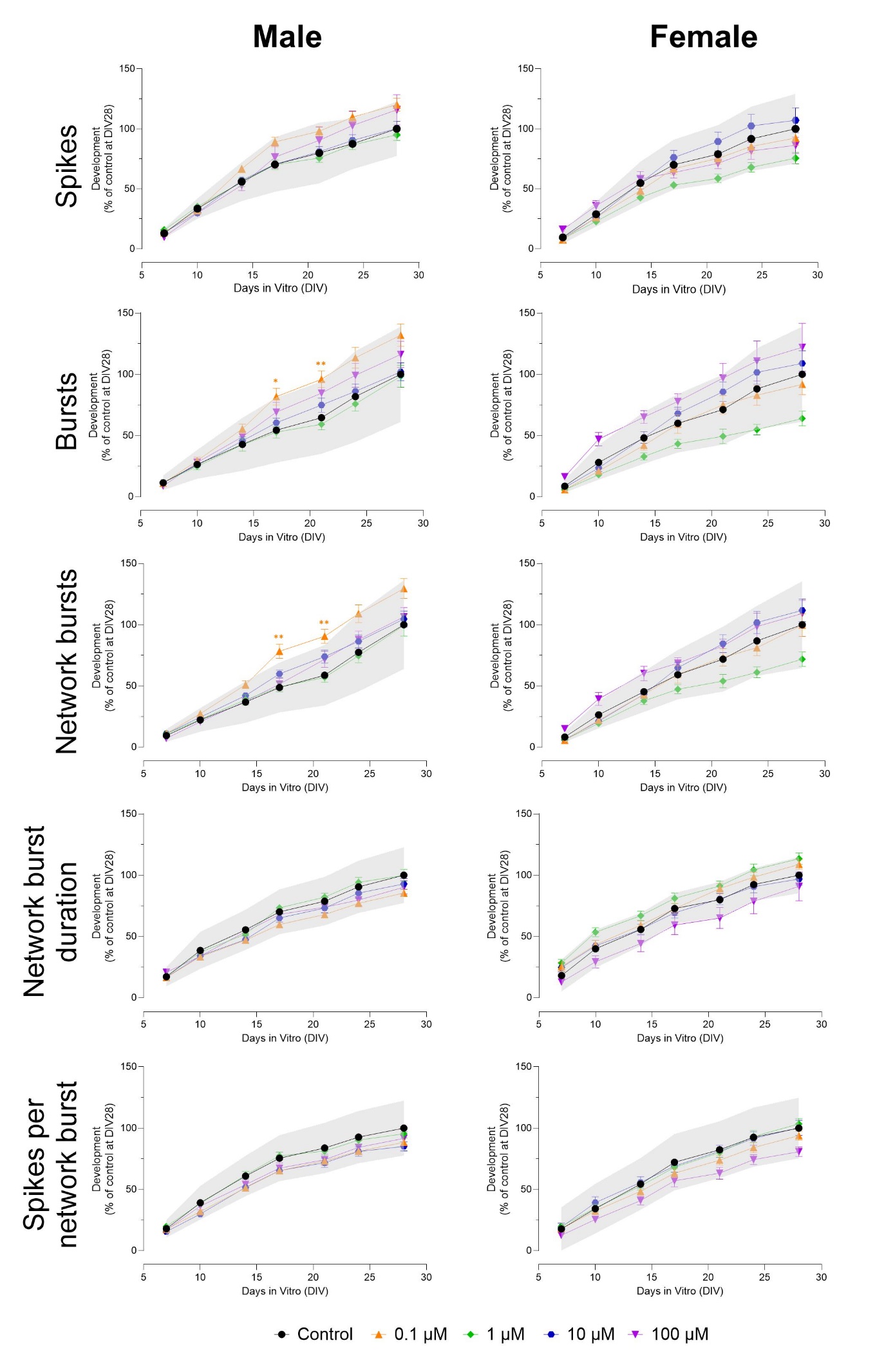


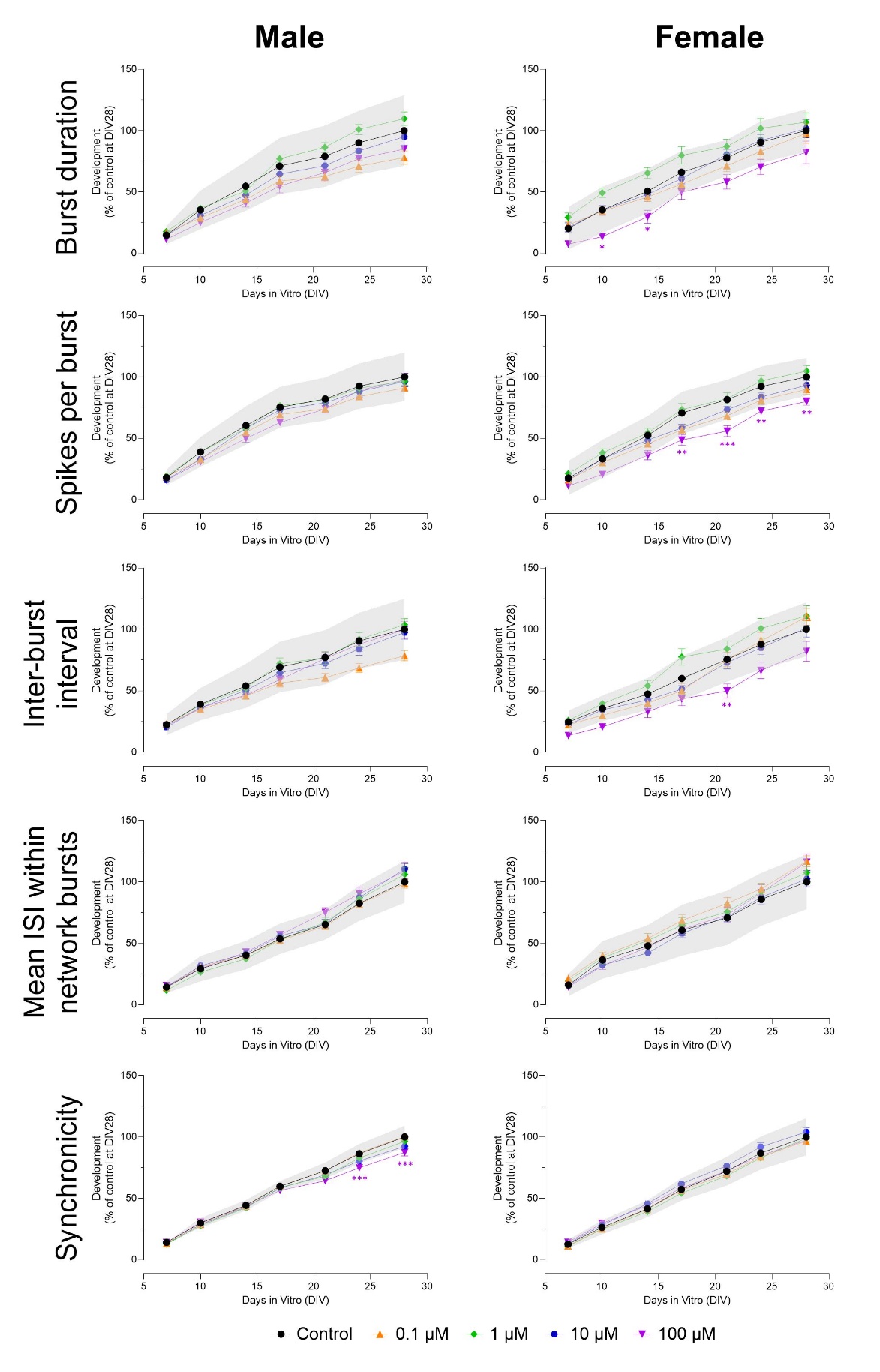
**Figure S14**. Effects of exposure to 3-phenoxybenzoic acid (3-PBA; DIV 7-28) on a subset of neuronal activity parameters in both male (left) and female (right) cultures. The grey shaded area represents a benchmark response derived from the variation in DMSO control experiments. Data points display average percentage compared to control (DMSO control at DIV 28 set to 100%) ± SEM from 7-40 individual wells (≥ 2 independent experiments per concentration). Difference from DMSO control (* *p*≤0.05; ** *p*≤0.01; *** *p*≤0.001). Color of asterisks indicates which concentration is significantly affected.

**Figure S15.** Exposure (DIV 7-28) to 0.1-100 µM permethrin (A), alpha-cypermethrin (B), and 3-PBA (C) does not lead to changes in cell viability. The grey shaded area represents a benchmark response of 15%, which is derived from the average variation in DMSO control experiments. Bars represent the average percentage viability (± SD; ≥ 10 wells from ≥ 2 independent experiments) compared to control
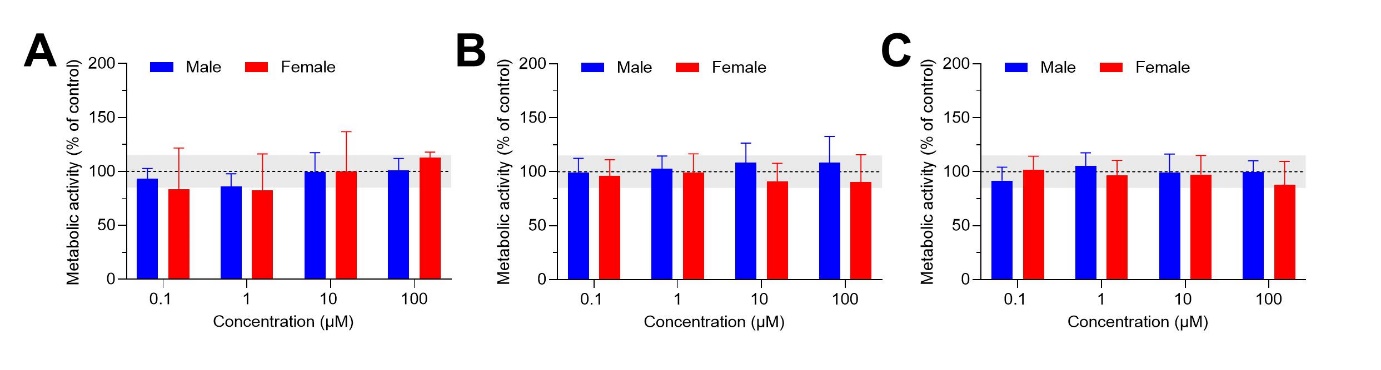
.
